# The COVIRL002 Trial-Tocilizumab for management of severe, non-critical COVID-19 infection: A structured summary of a study protocol for a randomised controlled trial

**DOI:** 10.1186/s13063-020-04680-w

**Published:** 2020-09-03

**Authors:** Aoife Cotter, Deborah Wallace, Cormac McCarthy, Eoin Feeney, Lorraine O’Neill, John Stack, Geraldine McCarthy, Rabia Hussain, Elena Alvarez Barco, Peter Doran, Patrick Mallon

**Affiliations:** 1 Mater Misericordiae University Hospital, Dublin, Ireland; 2 School of Medicine, University College Dublin, Dublin, Ireland; 3 St Vincent’s University Hospital, Dublin, Ireland; 4 HRB Trial Methodology Research Network, Galway, Ireland

**Keywords:** COVID-19, Randomised controlled trial, protocol, Tocilizumab, Immunology, Therapeutics

## Abstract

**Objectives:**

Tocilizumab is a humanized monoclonal antibody which targets and inhibits interleukin-6 (IL-6) and has demonstrated efficacy in treating diseases associated with hyper-inflammation. Data are suggestive of tocilizumab as a potential treatment for patients with COVID-19 infection. The aim of this study is to determine the safety and efficacy of standard dose versus low dose tocilizumab in adults with severe, non-critical, PCR-confirmed COVID-19 infection with evidence of progressive decline in respiratory function and evolving systemic inflammation on time to intubation, non-invasive ventilation and/or all-cause mortality.

**Trial design:**

This trial is a phase 2, open label, two-stage, multicentre, randomised trial.

**Participants:**

Adult subjects with severe, non-critical, PCR-confirmed COVID-19 infection with evidence of progressive decline in respiratory function and evolving systemic inflammation requiring admission to hospital at St. Vincent’s University Hospital and Mater Misericordiae University Hospital, Dublin, Ireland. *Inclusion criteria* Aged 18 years or older. Confirmed SARS-CoV2 infection (as defined by positive PCR). Evidence of hyper inflammatory state as evidenced by at least three of the following: Documented temperature >38°C in the past 48 hours, IL6 >40 pg/ml, or in its absence D-dimer >1.5 μgFEU /ml, Elevated CRP (>100mg/L) and/or a three-fold increase since presentation, Elevated ferritin X5 ULN, Elevated LDH (above the ULN), Elevated fibrinogen (above the ULN). Pulmonary infiltrates on chest imaging. Moderate to severe respiratory failure as defined by PaO_2_/FiO_2_≤300mmHg.

**Intervention and comparator:**

Intervention for participants in this trial is SOC plus Tocilizumab compared to SOC alone (comparator). For Stage 1, following randomisation, subjects will receive either (Arm 1) SOC alone or (Arm 2) SOC plus Tocilizumab (standard single dose – 8mg/kg, infused over 60 minutes. Once stage 1 has fully recruited, subsequent participants will be enrolled directly into Stage 2 and receive either (Arm 1) SOC plus Tocilizumab (standard single dose – 8mg/kg, infused over 60 minutes or (Arm 2) SOC plus Tocilizumab (standard single dose – 4mg/kg, infused over 60 minutes).

**Main outcomes:**

The primary endpoint for this study is the time to a composite primary endpoint of progression to intubation and ventilation, non-invasive ventilation or death within 28 days post randomisation.

**Randomisation:**

Eligible patients will be randomised (1:1) using a central register. Randomisation will be performed through an interactive, web-based electronic data capturing database. In stage 1, eligible participants will be randomised (1:1) to (Arm 1) SOC alone or to (Arm 2) SOC with single dose (8mg/kg, maximum 800mg) intravenous tocilizumab infused over 60 minutes.

In stage 2, eligible participants will be randomised (1:1) to receive either (Arm 1) single, standard dose (8mg/kg, maximum 800mg) intravenous tocilizumab infused over 60 minutes or (Arm 2) reduced dose (4mg/kg, maximum 800mg) intravenous tocilizumab infused over 60 minutes.

**Blinding:**

This study is open label. The study will not be blinded to investigators, subjects, or medical or nursing staff. The trial statistician will be blinded for data analysis and will be kept unaware of treatment group assignments. To facilitate this, the randomisation schedule will be drawn up by an independent statistician and objective criteria were defined for the primary outcome to minimize potential bias.

**Numbers to be randomised:**

In stage 1, 90 subjects will be randomised 1:1, 45 to SOC and 45 subjects to SOC plus Tocilizumab (8mg/kg, infused over 60 minutes). In stage 2, sample size calculation for the dose evaluation stage will use data generated from stage 1 using the same primary endpoint as in stage 1.

**Trial Status:**

The COVIRL002 trial (Protocol version 1.4, 13^th^ May 2020) commenced in May 2020 at St. Vincent’s University Hospital and Mater Misericordiae University Hospital, Dublin, Ireland. Recruitment is proceeding with the aim to achieve the target sample size on or before April 2021.

**Trial registration:**

COVIRL002 was registered 25 June 2020 under EudraCT number: 2020-001767-86 and Protocol identification: UCDCRC/20/02.

**Full protocol:**

The full protocol for COVIRL002 is attached as an additional file, accessible from the Trials website (Additional file 1). In the interest in expediting dissemination of this material, the familiar formatting has been eliminated; this Letter serves as a summary of the key elements of the full protocol.

The study protocol has been reported in accordance with the Standard Protocol Items: Recommendations for Clinical Interventional Trials (SPIRIT) guidelines (Additional file 2).

## Supplementary information

**Additional file 1.** Full Study Protocol.

**Additional file 2.** SPIRIT 2013 Checklist: Recommended items to address in a clinical trial protocol and related documents.

## Data Availability

Individual requests for access to the trial database will be considered in discussion with the local Research Ethics Committee.

