# Supplementary material for: The COVIRL002 Trial-Tocilizumab for management of severe, non-critical COVID-19 infection: A structured summary of a study protocol for a randomised controlled trial
Source: Trials. 2020 Sep 3;21:758. doi: 10.1186/s13063-020-04680-w (PMC7468092; doi:10.1186/s13063-020-04680-w)
Supplement: Supplementary file 1 — Additional file 1. Full Study Protocol. [file 13063_2020_4680_MOESM1_ESM.pdf]

COVID-19 Pre-ICU Trials Ireland

Treatment Study 002

Tocilizumab for management of severe, non-critical COVID-19 infection

---

An open-label, multi-centre, randomised trial comparing different doses of single-dose tocilizumab in adults with severe, non-critical, PCR-confirmed COVID-19 infection with evidence of progressive decline in respiratory function and evolving systemic inflammation on time to intubation, non-invasive ventilation and/or all-cause mortality

---

Protocol date: 13th May 2020

Protocol version: 1.4

EudraCT number: **2020-001767-86**

Sponsor: University College Dublin

Sponsor's Protocol Code: UCDCRC/20/02

Chief Investigators: Prof Patrick Mallon[1,2]
Prof of Microbial Diseases,
Consultant Infectious Diseases
Tel: +353 1 716 5821
Email: Paddy.mallon@ucd.ie

Site Principal Investigators: Dr Eoin Feeney[1,2]
Consultant Infectious Diseases
St Vincent's University Hospital
Email: eoin.feeney@svuh.ie

Dr Aoife Cotter[1,3]
Consultant Infectious Diseases
Mater Miseroicordiae University Hospital
Email: aoife.cotter@ucd.ie

Project Leader: Elena Alvarez Barco[1]

Laboratory Leader: Alejandro Garcia-Leon[1]

Study Statistician: Willard Tinago[1]

Co-Investigators:

St Vincent's University Hospital
Cormac McCarthy
Silke Ryan
Lorraine O'Neill
Alistair Nicholl
Marcus Butler
Charles Gallagher
Sarmad Waqas
Cathal O'Brion
Stefano Savinelli

Mater Misericordiae University Hospital
Tara McGinty
Eavan Muldoon
Jack Lambert
Gerard Sheehan
Geraldine McCarthy
John Stack
Jim Egan
Sean Gaine
Brian McCullough
Dermot O'Callaghan

University College Dublin

Peter Doran, Scientific Director UCD Clinical Research Centre

Study sites:

1. UCD Centre for Experimental Pathogen Host Research (CEPHR)
School of Medicine, Belfield,
Dublin 4, Dublin, Ireland

2. St Vincent's University Hospital (SVUH)
Merrion Road, Dublin 4, Dublin, Ireland

3. Mater Misericordiae University Hospital
Eccles Street, Dublin 7, Dublin, Ireland

PROTOCOL SIGNATURE PAGE

**Protocol Title:**	An open-label, multi-centre, randomised trial comparing different doses of single-dose tocilizumab in adults with severe, non-critical, PCR-confirmed COVID-19 infection with evidence of progressive decline in respiratory function and evolving systemic inflammation on time to intubation, non-invasive ventilation and/or all-cause mortality
**Protocol Number:**	UCDCRC/20/02
**Protocol Version/ Date:**	Version 1.4, 13-May-2020
**Sponsor Name:**	University College Dublin

I, the undersigned, am responsible for the conduct of the trial at this site and agree to the following:

- I understand and will conduct the trial according to the protocol, any approved protocol amendments, ICH GCP and all applicable regulatory authority requirements and national laws.
- I will not deviate from the protocol without prior written permission from the Sponsor and prior review and written approval from Independent Ethics Committee, except where necessary to prevent any immediate danger to the subject.
- I have read and understand fully the SmPCs for the investigational medicinal products; and I am familiar with the Investigational Medicinal Product(s) (IMP) and its use according to this protocol.
- I have sufficient time to properly conduct and complete the trial within the agreed trial period, and I have available an adequate number of qualified staff and adequate facilities for the foreseen duration of the trial to conduct the trial properly and safely.
- I will ensure that any staff at my site(s) who are involved in the trial conduct are adequately trained regarding the Investigational Medicinal Products, the protocol and their responsibilities. In the case of delegating any of my trial responsibilities I will provide the Sponsor with a Delegation of Activities certificate.

	Name	Signature	Date
**SPONSOR**	**Prof Peter Doran**	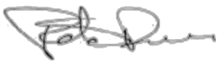	13-May-2020
**CHIEF INVESTIGATOR**	**Prof Patrick Mallon**	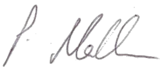	13-May-2020
**SITE PRINCIPAL INVESTIGATOR**			

Document History

Document	Date of Issue	Summary of Change
Version 1.0 - Original protocol	09-Apr-2020	Not applicable
Version 1.1	21-Apr-2020	Amendment in response to regulatory and ethical reviews including:<br>- Body weight ≤ 30kg added as an exclusion criterion<br>- Section 4 – Assessment of Safety – Full definition for an SAE provided.<br>- Section 4.1.2 – Assessment of Causality - additional note that all AEs will be captured and reported.<br>- Section 5.1.2 – Record Retention - Essential documents will be retained for 15 years<br>- Section 8 – Data Safety Monitoring Board – additional detail provided.
Version 1.2	26-Apr-2020	Amendment in response to regulatory and ethical reviews including:<br>- Updated detail in statistical section
Version 1.3	03-May-2020	Amendment in response to regulatory reviews including:<br>- Section 8 – Data Safety Monitoring Board – additional detail provided<br>- Section 2.2 Trial design – additional data provided
Version 1.4	13-May-2020	Amendment in response to regulatory reviews including:<br>- Section 8 – Data and Safety Monitoring Board – additional detail provided

Abbreviations

AE	Adverse Event
AST	Aspartate aminotransferase
ALT	Alanine transaminase
BID	Twice daily
CRF	Case report form
CRP	C-reactive protein
CXR	Chest X-ray
CDAD	Clostridium difficile-associated diarrhoea
DSMB	Data Safety Monitoring Board
ECG	Electrocardiogram
EDTA	Ethylenediaminetetraacetic acid
G6PD	Glucose-6-phosphate dehydrogenase
GCP	Good Clinical Practice
HCQ	Hydroxychloroquine
HPRA	Health Products Regulatory Authority
HRCDC	Health Research Consent Declaration Committee
ICU	Intensive Care Unit
IEC	Independent Ethics Committee
IMP	Investigational Medicinal Product
IRB	Independent Review Board
LDH	Lactate dehydrogenase
PIS	Patient Information Statement
PCR	Polymerase chain reaction
SAE	Serious Adverse Event
SAR	Serious Adverse Reaction
SOP	Standard operating procedure
SPC/SmPC	Summary of Product Characteristics
SUSAR	Suspected Unexpected Serious Adverse Reaction

Protocol Synopsis

Title	Tocilizumab for management of severe, non-critical COVID-19 infection
Protocol Number	Version 1.4
Objectives	Primary objective:<br><br>To determine the safety and efficacy of standard dose versus low dose tocilizumab in adults with severe, non-critical, PCR-confirmed COVID-19 infection with evidence of progressive decline in respiratory function and evolving systemic inflammation on time to intubation, non-invasive ventilation and/or all-cause mortality.<br><br>Secondary objectives:<br><br>In adults with severe, non-critical, PCR-confirmed COVID-19 infection with evidence of progressive decline in respiratory function and evolving systemic inflammation, to determine if single dose administration of tocilizumab will be safe, as defined by no difference in all-cause mortality compared to standard of care.<br><br>In adults with severe, non-critical, PCR-confirmed COVID-19 infection with evidence of progressive decline in respiratory function and evolving systemic inflammation, to determine if single dose administration of tocilizumab will result in a longer time to intubation and non-invasive ventilation and lower all-cause mortality than standard of care.<br><br>In adults with severe, non-critical, PCR-confirmed COVID-19 infection with evidence of progressive decline in respiratory function and evolving systemic inflammation, to determine if, compared to standard dose, single dose administration of tocilizumab at 50% of the standard dose will result in similar time to intubation and non-invasive ventilation and all-cause mortality than standard of care.
Study Design	Phase 2, open label, two-stage, multicentre, randomised trial.<br><br>In stage 1, eligible participants will be randomised (1:1) to receive either standard of care alone or standard of care plus single dose (8mg/kg, maximum 800mg) intravenous tocilizumab infused over 60 minutes.<br><br>Following completion of stage 1, should tocilizumab be shown to be safe, in stage 2, eligible participants will be randomised 1:1 to receive either single, standard dose (8mg/kg, maximum 800mg) intravenous tocilizumab infused over 60 minutes or reduced dose (4mg/kg, maximum 800mg) intravenous tocilizumab infused over 60 minutes.
Patient Population and sample size	Adults with PCR-confirmed COVID-19 infection requiring admission to hospital.<br><br>Inclusion criteria:<br>• Confirmed SARS-CoV2 infection (as defined by positive PCR)<br>• Evidence of hyper inflammatory state as evidenced by at least three of the following<br>o Documented temperature >38°C in the past 48 hours

	o IL6 >40 pg/ml, or in its absence D-dimer >1.5 µgFEU /ml.<br>o Elevated CRP (>100mg/L) and/or a three-fold increase since presentation<br>o Elevated ferritin X5 ULN<br>o Elevated LDH (above the ULN)<br>o Elevated fibrinogen (above the ULN)<br>• Pulmonary infiltrates on chest imaging<br>• Moderate to severe respiratory failure as defined by $PaO_2/FiO_2 \leq 300mmHg$<br>• Aged 18 years or older<br><br>Exclusion Criteria<br>• Primary or secondary immunodeficiency<br>• Use of significant immunosuppressive therapy in the last 3 months (not including hydroxychloroquine or short course of corticosteroids (defined as <400mg cumulative dose)<br>• Active malignancy requiring treatment<br>• Known active current or history of recurrent bacterial, mycobacterial, fungal or viral infections including history of untreated latent TB<br>• History of diverticulitis or chronic ulcerative GI disease that might predispose to GI perforation<br>• Severe allergic reaction to monoclonal antibodies<br>• Pregnancy or breast feeding<br>• AST / ALT with values greater than 10 times normal levels or history of significant liver disease that in the opinion of the investigator precludes use of an investigational agent<br>• Neutrophils < 0.5 $x10^9/L$<br>• Platelets < $50x10^9/L$<br>• Documented, uncontrolled sepsis caused by pathogen(s) other than COVID-19<br>• Presence of co-morbidities (including cognitive impairment and/or frailty) that, in the opinion of the investigator, should preclude use of an investigational agent<br>• Current skin or soft tissue infection not controlled by antibiotics<br>• Body weight ≤ 30kg
Sample size	In stage 1, we will recruit 90 subjects (45 to each arm). We estimated that 86 patients (43 assigned to standard of care and to single dose 8mg/kg intravenous tocilizumab) would provide 80% power to detect a decrease in the proportion with composite endpoint of intubation and ventilation, non-invasive ventilation or death at day 8 from 25% with SOC to 10% with SOC + tocilizumab (HR=0.37) with a 5% type-1 error.<br><br>A single interim analysis is planned at information fractions of 50% (16 out of 32 events) with α-spending following the Lan-DeMets spending function with an O'Brien-Fleming approach for efficacy, with little impact on the final sample size. Similarly futility will be planned to coincide with the efficacy interim analysis using an O'Brien–Fleming–like type II error spending function for monitoring. At the interim analysis, the primary endpoint will be assessed and enrolment will only stop at an interim analysis if the endpoint meets pre-specified stopping criteria for efficacy. Although the calculation incudes a

	possible interruption due to futility, this can be ignored, at the discretion of the DSMB without any impact on the type I error.<br><br>In stage 2, sample size calculation for the dose evaluation stage will use data generated from stage 1, using the same primary endpoint as in Stage 1. Final sample size will be determined and implemented through a protocol amendment.
Period of study	April 2020 to April 2021
Study assessments	Both stage 1 and stage 2 studies will comprise seven study visits over 28 days post randomisation.<br><br>Following informed consent and screening for eligibility, participants will be recruited only to stage 1. Once stage 1 has fully recruited, subsequent participants will be enrolled directly to stage 2.<br><br>The screening visit may occur on same day as baseline visit. At the baseline visit subjects will be randomised to the following arms:<br><br>Stage 1.<br>Arm 1. Standard of care<br>Arm 2. Standard of care plus single dose (8mg/kg, maximum 800mg) intravenous tocilizumab infused over 60 minutes.<br><br>Stage 2.<br>Arm 1. Standard of care plus single, standard dose (8mg/kg, maximum 800mg) intravenous tocilizumab infused over 60 minutes<br>Arm 2. Standard of care plus single, reduced dose (4mg/kg, maximum 800mg) intravenous tocilizumab infused over 60 minutes<br><br>The following assessments will be performed at study visits as set out in the Schedule of Visits:<br><br>- Assessment of safety (AE / SAE).<br>- Clinical assessment of respiratory status and symptom-directed physical examination.<br>- Bloods for haematology, clinical chemistry, inflammatory markers (IL6 and/or D-dimer, CRP, ferritin, fibrinogen, troponin, LDH) and arterial blood gas (if clinically indicated)<br>- Pregnancy test (screening only in female subjects)<br>- Sample storage (serum, plasma, PBMC, urine, stool and respiratory samples (nasopharyngeal swabs and/or sputum))<br>- Hepatitis and HIV serology and quantiferon (results will not be used to determine eligibility)<br>- ECG<br>- Chest x-ray
Endpoints	Stage 1<br><br>Primary endpoint:<br>• Time to a composite primary endpoint of progression to intubation and ventilation, non-invasive ventilation or death<br><br>Secondary endpoints:<br>• Prevalence of new SAE at day 8<br>• Time to a composite primary endpoint of progression to intubation and ventilation, non-invasive ventilation or death at 8, 14 and 28 days<br>• Survival at 8, 14 and 28 days

<table>
<tr><td></td><td>

Incidence of intercurrent bacterial sepsis (positive blood culture) or septic shock (regardless of causative agent)
Change from baseline to day 8, 14 and 28 in markers of inflammation (CRP, ferritin, D-dimer, LDH and IL6)
Time to PCR negativity and change from baseline in quantitative SARS-CoV-2 viral load (measured by CT values)
Time to PCR negativity from date of onset of symptoms

Stage 2

Primary endpoint:

Time to composite primary endpoint of intubation and ventilation, non-invasive ventilation or death

Secondary endpoints:

Prevalence of new SAE over the follow-up of the study
Time to composite primary endpoint of intubation and ventilation, non-invasive ventilation or death at 8, 14 and 28 days
Survival at 8, 14 and 28 days
Incidence of intercurrent bacterial sepsis (positive blood culture) or septic shock (regardless of causative agent)
Change in oxygen requirements and severity of respiratory failure as measured by change in PaO_2/FiO_2
Change in Aa-gradient
Change from baseline in markers of inflammation

</td></tr>
<tr><td>Data analysis</td><td>
Stage 1 Analysis:

Analysis of efficacy endpoints will be done on the intention-to-treat population, and safety in all patients who received at least one dose of single dose of intravenous tocilizumab or standard of care. Categorical will be summarized using counts and proportions and continuous data using mean (standard deviation) or median (interquartile range), as appropriate. The composite primary-endpoint (time to intubation and ventilation, non-invasive ventilation or death) will be estimated using Kaplan-Meier methods and compared between treatment groups with the log-rank test. Hazard ratios for the composite primary endpoint and 95% confidence intervals (CIs) comparing the treatment arms will be estimated using Cox proportional hazards models. Exploratory analyses on change in secondary continuous end-points from baseline to 28 days after randomisation will be compared between the two treatments groups by analysis of covariance (ANCOVA) adjusted for baseline values. Where appropriate, continuous outcomes endpoints may be log-transformed prior to analysis. Time to event secondary endpoints will be explored using a similar approach to the primary endpoint.

Adverse event (AE) term recorded during the study will be mapped to a system organ class and preferred term using the current MedDRA Dictionary (Version 10.0) or later compared using the chi-square tests. A post-hoc procedure to account for multiplicity in tests of secondary end-points, with a Bonferroni-adjusted significance level will applied to any claim for benefit in the secondary end points.

Phase 2
</td></tr>
</table>

	Sample size calculation for this dose evaluation stage will use data generated using the same primary endpoint as in Stage 1. We propose using a 2-stage design proposed by Sin-Ho Jung to evaluate which dosage of single dose (8mg/kg vs 4mg/kg) intravenous tocilizumab in combination with standard is efficacious in the treatment of patients with severe, non-critical, PCR-confirmed COVID-19 infection to warrant further investigation.<br><br>To establish efficacy, differences in composite endpoint event rates between arms will be evaluated using the criteria propose by Sin-Ho Jung. Exploratory evaluation of the primary and secondary endpoints will be similar to those used in the analysis in Phase 1 of the study.

Contents:

Abbreviations....5
Protocol Synopsis....6
1 Introduction....15
1.1 Study objectives....17
1.1.1 Primary objective....17
1.1.2 Secondary objectives....17
2 Trial Design....17
2.1 Design....17
2.2 Trial Plan....17
2.3 Source of subjects....18
2.4 Number of Subjects....18
2.5 Inclusion criteria....19
2.6 Exclusion criteria....19
3 Trial Methods....19
3.1 Investigational medicinal product....19
3.1.1 Method of Administration....20
3.1.2 Special warnings and precautions for use....20
3.1.3 Interactions with other medicinal products....21
3.1.4 More common undesirable effects....21
3.1.5 Overdose....22
3.2 Supply, Packaging and Storage....22
3.3 Informed Consent:....22
3.4 Inclusion/Exclusion Criteria:....23
3.5 Inclusion criteria....23
3.6 Exclusion criteria....23
3.7 Clinical and medication history....24
3.7.1 Demographics:....24
3.7.2 COVID-19 History:....24
3.7.3 Medical History:....24
3.7.4 Concomitant medications:....24
3.8 Screening Assessments / Procedures....25
3.8.1 Physical Examination....25
3.8.2 Vital signs....25
3.8.3 ECG....25
3.8.4 Chest X-ray:....25
3.8.5 Pregnancy test:....25
3.8.6 Adverse events:....25
3.9 Assignment of Study Number....25
3.10 Randomisation....26
3.11 Clinical assessments / Procedures....26
3.11.1 Screening:....26
3.11.2 Baseline visit (Day 0):....27
3.11.3 Day 1 visit:....27
3.11.4 Day 2 visit:....28
3.11.5 Day 4 visit:....28
3.11.6 Day 8 visit....28
3.11.7 Day 14 visit....29
3.11.8 Day 28 visit....29
3.12 Laboratory assessments....29
3.12.1 Laboratory Assessments:....29
3.12.2 Stored samples....30
4 Assessment of safety....30
4.1 Evaluation of AEs and SAEs....30
4.1.1 Assessment of seriousness....31
4.1.2 Assessment of causality....31

4.1.3 Assessment of severity 32
4.1.4 Assessment of expectedness 32
4.1.5 Relation to Study IMP 32
4.2 Procedures for Recording and Reporting Serious Adverse Events 32
4.3 Sponsor Responsibilities: 33
4.4 Procedures for Documenting and Reporting Pregnancies 33
5 Statistical plan and Interim analysis 33
6 DATA MANAGEMENT 34
6.1 Data Quality Assurance 35
6.2 Record Retention 35
6.3 Confidentiality of Subject Records 35
6.4 Confidentiality of Study Data 35
7 DATA AND SAFETY MONITORING BOARD 36
8 REGULATORY APPROVAL 36
9 QUALITY CONTROL & QUALITY ASSURANCE PROCEDURES 37
10 FINANCING & INSURANCE/INDEMNITY 38
11 Bibliography 39
12 Appendices 40
Appendix A – Tocilizumab Summary of Product Characteristics – SmPC 40
Appendix B- Product Labelling 81
Appendix C- Sample Storage 82
Appendix D- Laboratory Reference Ranges 83
Appendix E - Instructions for measuring height and weight. 86
Appendix F - Instructions for measuring hip and waist circumferences. 87
Appendix G – Adverse Events and Serious Adverse Events. 88
Appendix H - Declaration of Helsinki 89

Schedule of Study Visits and Procedures

Study visit	Screening	Baseline (Day 0)	Day 1	Day 2	Day 4	Day 8 (± 1)	Day 14 (± 2)	Day 28 (±3)
Administrative procedures								
Informed consent	X							
Randomisation		X						
Dispense study medication		X						
Clinical/Medical assessments								
Demographic, clinical and medical history[1]	X							
Medication review	X							
Assessment of Safety		X	X	X	X	X	X	X
Full physical examination (including BMI)	X							
Directed physical exam		X	X	X	X	X	X	X
Vital signs	X	X	X	X	X	X	X	X
ECG	X[2]							
Chest X-ray	X[2]			X	X	X	X[3]	X[3]
Laboratory Assessments								
Swab for COVID-19 PCR	X[4]					X	X[5]	
Quantiferon		X						
HIV and Hepatitis serology[12]		X						
FBC and differential	X[6]	X[7]	X	X	X	X	X[9]	X[9]
Chemistry panel (Liver, renal and bone profile) and CK[8]	X[6]	X[7]	X	X	X	X	X[9]	X[9]
Lipids[11]	X					X		
HbA1C	X							
Ferritin	X[6]	X[7]	X	X	X	X	X	X
Fibrinogen	X[6]	X[7]	X	X	X	X	X	X
HbA1c	X							X
Troponin	X[6]	X[7]	X	X	X	X	X	X
IL6 and/or D-dimer	X[6]	X[7]	X	X	X	X	X	X
CRP	X[6]	X[7]	X	X	X	X	X	X
LDH	X[6]	X[7]	X	X	X	X	X	X

Arterial blood gas		X	X[9]	X[9]	X[9]	X	X[9]	
Pregnancy test (serum or urine βHCG)[10]	X							
Sample Storage								
Serum/plasma/PBMC samples		X		X		X	X	X
Urine / stool samples		X				X	X	X
Respiratory samples		X		X		X	X	X

1. Including record of most recent chest x-ray findings along with most recent blood tests (FBC, urea, creatinine, AST, ALT)
2. If not performed in previous 36 hours
3. If residual changes observed on previous CXR
4. If a positive result is not available from the current inpatient admission
5. If swab from Day 8 returns a positive result
6. If not performed in previous 24 hours
7. Only to be performed if screening visit and baseline visit **NOT** performed on same day.
8. Bilirubin, alkaline phosphatase, alanine aminotransferase, gamma glutamyl transferase, albumin; urea, creatinine; corrected calcium and phosphate, creatinine kinase
9. Only to be performed if clinically indicated
10. For women of child-bearing potential or if pregnancy is suspected. If a urine pregnancy test is positive, it must be confirmed with a serum pregnancy test. A woman is considered of childbearing potential (WOCBP), i.e. fertile, following menarche and until becoming post-menopausal unless permanently sterile. Permanent sterilisation methods include hysterectomy, bilateral salpingectomy and bilateral oophorectomy.
11. Lipids include total cholesterol, HDL cholesterol, LDL cholesterol and triglycerides. Can be fasting or non-fasting
12. Hepatitis B serology to include hepatitis B sAg, cAb and sAb

1 Introduction

On 31 December 2019, China reported cases of pneumonia from a previously unknown coronavirus, 2019-nCoV, also called SARS-CoV-2. The disease it causes has been called COVID-19. The World Health Organization (WHO) designated COVID-19 a “public health emergency of international concern” on 30 January 2020 and declared it a pandemic on 11 March 2020. Coronaviruses are single-stranded RNA viruses that encode for four enzymes essential to the viral life cycle. They enter mammalian cells through an interaction of a viral spike glycoprotein and a receptor angiotensin 1 converting enzyme 2 (ACE2) for SARS-CoV-1 and SARS-CoV-2 and dipetidylpeptidase 4 for MERS. Attempts to develop effective treatments against two other coronavirus diseases, SARS and MERS, have so far been unsuccessful.

In some individuals, infection with SARS-CoV2 results in a severe form of COVID-19 disease characterised by a progressive hyperinflammatory state accompanied by viral pneumonitis leading to hypoxia and in some cases respiratory failure requiring ventilatory support. Mortality in ventilated patients suffering from severe COVID-19 infection has been reported to be as high as 62% (https://www.ncbi.nlm.nih.gov/pubmed/32105632).

Tocilizumab is a humanized monoclonal antibody which targets and inhibits interlukin-6 (IL-6), a pro-inflammatory cytokine implicated in the pathogenesis of several autoimmune diseases [1]. Most commonly it is used in the treatment of rheumatoid arthritis and giant cell arteritis where it has become an established treatment option for patients with refractory and/or severe disease [2, 3]. There is randomised control trial (RCT) evidence demonstrating tocilizumab efficacy in the treatment of systemic juvenile inflammatory arthritis (sJIA) and case series evidence demonstrating efficacy in the treatment of adult onset stills disease (AOSD) [1]. Both sJIA and AOSD can progress to secondary haemophagocytic lymphohistiocytosis (sHLH), a hyper-inflammatory syndrome characterised by cytokine release, multi-organ failure and high mortality [4]. Cases of tocilizumab effectively treating sHLH have been reported [5].

Tocilizumab is licensed for the treatment of cytokine release syndrome (CRS), a side-effect of CAR-T cell immunotherapy [6, 7]. Both sHLH and CRS share similar features and are felt to lie within the same spectrum of hyper-inflammatory disease. Tocilizumab has therefore to some degree, demonstrated efficacy in treating diseases associated with hyper-inflammation.

<u>Rationale for use of Tocilizumab in COVID-19 associated hyper-inflammation:</u>

Evidence has emerged from the COVID-19 pandemic literature that a subset of patients with COVID-19 disease express clinical and biochemical features of hyperinflammation with increased levels of interleukin (IL), -2, -6 -7, -8, granulocyte-colony stimulating factor, interferon-γ inducible protein 10, monocyte chemoattractant protein 1, macrophage inflammatory protein 1-α and tumour necrosis factor-α [8]. Predictors of fatality from two recent retrospective, multi-centre studies of confirmed COVID-19 cases included elevated ferritin, c-reactive protein (CRP) and serum IL-6, which are also biomarkers of hyper-inflammation [9, 10].

These observations led investigators in China to treat a group of 21 patients with COVID-19 infection and markers of hyper- inflammation with tocilizumab. Following tocilizumab treatment all patients became afebrile within 24 hours. A reduction of oxygen requirement was observed (15/20 patients), resolution of CT lesions (19/21 patients), normalization of lymphocyte count (10/19 patients), reduction of CRP levels (16/19 patients) and hospital discharge (19/21 patients) with an average hospitalization duration of 13.5 days [11]. There were no adverse events attributable to tocilizumab. The major caveat is that this was a small, retrospective, open label study with no control arm. Larger, prospective multi-centre randomised controlled trials are currently taking place in in China (ChiCTR2000029765) and in Italy (EudraCT 2020-001110-38). Compassionate use of

tocilizumab has become widespread practice in Italy and other European countries in patients with severe COVID-19 disease, despite lack of RCT evidence.

Dosing:

Tocilizumab can be administered via intravenous or subcutaneous routes. In RA, tocilizumab given via IV and SC routes has been shown to be equivocal in terms of safety and efficacy [12]. The licenced dose of tocilizumab for CRS associated with CAR-T therapy is 8mg/kg IV, every 8 hours, maximum of 4 doses [7]. There is no licenced dose for tocilizumab in COVID-19 disease but in clinical practice it is given as 8mg/kg IV as a STAT dose, repeated after 12 hours if there is no response.

Risk and Benefits

Potential Risks

The primary risks of this study are related to the potential adverse effects associated to the study drugs and study procedures.

The more common adverse effects associated with tocilizumab are described in the protocol within the Section 'Study Treatments' and a full list of undesirable effects is provided within Appendix A (Section 4.8 of Summary of Product Characteristics).

The risks associated with the study procedures include:

Blood sampling: This procedure may be uncomfortable but rarely results in any significant problems. Side-effects that have been noted with drawing blood include feeling light-headed or faint, fainting, formation of a blood clot, bruising and/or infection at the site of the needlestick.

Chest-X-ray (CXR): The radiation X-rays produces can harm living tissues. This risk is relatively small, but it increases with cumulative exposure. As part of everyday living, everyone is exposed to naturally occurring background radiation and receives a dose of about 2 to 3 milisieverts (mSv) per year. This study will involve exposure to a very small dose of radiation. The total effective dose from this study is of 0.4-0.7 mSv. At this level, no harmful effects of radiation have been demonstrated, and the risk is very low.

Electrocardiogram (ECG): ECGs are safe, non-invasive, painless tests and have no major risks. The electrodes that connect the sensors to the chest do not send out electric shocks. Some people may experience a skin rash where electrodes were placed. If any paste or gel was used to attach the electrodes, the subject may have an allergic reaction to it. The irritation usually goes away once the patches are removed without requiring treatment.

There are no identifiable psychological, sociological, economical or legal risks to the participants.

Potential Benefits

So far, an effective treatment against COVID-19 is yet to be found. Preliminary studies have suggested that tocilizumab may be a potential pharmacological agent for the treatment of COVID-19 infection. The information gained from this study may be of benefit in the treatment of other people infected with COVID-19 and in the understanding of markers of disease progression.

1.1 Study objectives

1.1.1 Primary objective

To determine the safety and efficacy of standard versus low dose tocilizumab in adults with severe, non-critical, PCR-confirmed COVID-19 infection with evidence of progressive decline in respiratory function and evolving systemic inflammation on time to intubation, non-invasive ventilation and/or all-cause mortality.

1.1.2 Secondary objectives

In adults with severe, non-critical, PCR-confirmed COVID-19 infection with evidence of progressive decline in respiratory function and evolving systemic inflammation, to determine if single dose administration of tocilizumab will be safe, as defined by no difference in all-cause mortality compared to standard of care.

In adults with severe, non-critical, PCR-confirmed COVID-19 infection with evidence of progressive decline in respiratory function and evolving systemic inflammation, to determine if single dose administration of tocilizumab will result in a longer time to intubation and non-invasive ventilation and lower all-cause mortality than standard of care.

In adults with severe, non-critical, PCR-confirmed COVID-19 infection with evidence of progressive decline in respiratory function and evolving systemic inflammation, to determine if, compared to standard dose, single dose administration of tocilizumab at 50% of the standard dose will result in similar time to intubation and non-invasive ventilation and all-cause mortality than standard of care.

2 Trial Design

2.1 Design

Phase 2, open label, two-stage, multicentre, randomised trial.

In stage 1, eligible participants will be randomised (1:1) to receive either standard of care alone or standard of care plus single dose (8mg/kg, maximum 800mg) intravenous tocilizumab infused over 60 minutes.

Following completion of stage 1, should tocilizumab be shown to be safe, in stage 2, eligible participants will be randomised 1:1 to receive either single, standard dose (8mg/kg, maximum 800mg) intravenous tocilizumab infused over 60 minutes or reduced dose (4mg/kg, maximum 800mg) intravenous tocilizumab infused over 60 minutes.

2.2 Trial Plan

In stage 1, eligible participants will be randomised (1:1) to receive either standard of care or standard of care with addition of single dose (8mg/kg, maximum 800mg) intravenous tocilizumab infused over 60 minutes.

In stage 2, eligible participants will be randomised 1:1 to receive either standard of care along with single, standard dose (8mg/kg, maximum 800mg) intravenous tocilizumab infused over 60 minutes or standard of care plus reduced dose (4mg/kg, maximum 800mg) intravenous tocilizumab infused over 60 minutes.

If, in the opinion of the investigator, the participant may benefit from a second dose of tocilizumab the patient should be removed from the study and treatment provided as per the opinion of the investigator.

Figure 1. Trial plan.

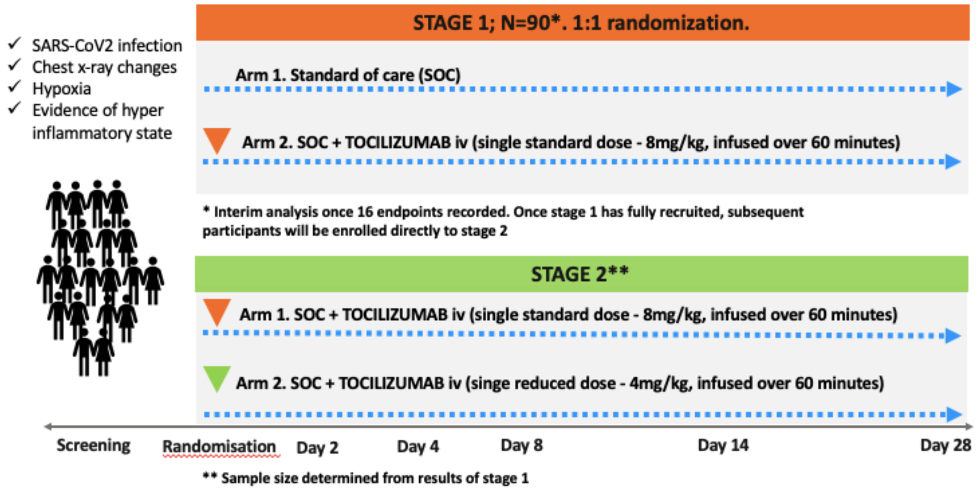

2.3 Source of subjects

Adult subjects will be recruited from participating clinical sites from those in-patients who are receiving care for PCR-confirmed COVID-19 infection.

2.4 Number of Subjects

In stage 1, we will recruit 90 subjects (45 to each arm). We estimated that 86 patients (43 assigned to standard of care and to single dose 8mg/kg intravenous tocilizumab) would provide 80% power to detect a decrease in the proportion with composite endpoint of intubation and ventilation, non-invasive ventilation or death at day 8 from 25% [13] with SOC to 10% with SOC + tocilizumab (HR=0.37) with a 5% type-1 error. Our sample size calculation is based on the fixed design, two-arm trial with time-to-event outcome by Lachin and Foulkes (1986) [14], with an accrual of 15 days and study duration of 28 days. Sample size calculations were computed using the *gsDesign* package in R (version 3.6.2).

A single interim analysis is planned at information fractions of 50% (16 out of 32 events) with α-spending following the Lan-DeMets spending function with an O'Brien-Fleming approach for efficacy, with little impact on the final sample size. Similarly futility will be planned to coincide with the efficacy interim analysis using an O'Brien–Fleming–like type II error spending function for monitoring. At the interim analysis, the primary endpoint will be assessed and enrolment will only stop at an interim analysis if the endpoint meets pre-specified stopping criteria for efficacy. Although the calculation incudes a possible interruption due to futility, this can be ignored, at the discretion of the DSMB without any impact on the type I error.

In stage 2, sample size calculation for the dose evaluation stage will use data generated from stage 1, using the same primary endpoint as in Stage 1. Final sample size will be determined and implemented through a protocol amendment.

In stage 2, we will conduct a 2-phase analytical approach proposed by Sin-Ho Jung [15] to evaluate which dosage of single dose (8mg/kg vs 4mg/kg) intravenous tocilizumab in combination with standard of care is efficacious in the treatment of patients with severe, non-critical, PCR-confirmed COVID-19 infection to warrant further investigation.

2.5 Inclusion criteria

- Aged 18 years or older
- Confirmed SARS-CoV2 infection (as defined by positive PCR)
- Evidence of hyper inflammatory state as evidenced by at least three of the following
  - Documented temperature >38°C in the past 48 hours
  - IL6 >40 pg/ml, or in its absence D-dimer >1.5 μgFEU /ml.
  - Elevated CRP (>100mg/L) and/or a three-fold increase since presentation
  - Elevated ferritin X5 ULN
  - Elevated LDH (above the ULN)
  - Elevated fibrinogen (above the ULN)
- Pulmonary infiltrates on chest imaging
- Moderate to severe respiratory failure as defined by $PaO_2/FiO_2 \leq 300mmHg$

2.6 Exclusion criteria

- Primary or secondary immunodeficiency
- Use of significant immunosuppressive therapy in the last 3 months (not including hydroxychloroquine or short course of corticosteroids (defined as <400mg cumulative dose)
- Active malignancy requiring treatment
- Known active current or history of recurrent bacterial, mycobacterial, fungal or viral infections including history of untreated latent TB
- History of diverticulitis or chronic ulcerative GI disease that might predispose to GI perforation
- Severe allergic reaction to monoclonal antibodies
- Pregnancy or breast feeding
- AST / ALT with values greater than 10 times normal levels or history of significant liver disease that in the opinion of the investigator precludes use of an investigational agent
- Neutrophils < 0.5 $x10^9$/L
- Platelets < 50$x10^9$/L
- Documented, uncontrolled sepsis caused by other pathogen(s) other than COVID-19
- Presence of co-morbidities (including cognitive impairment and/or frailty) that, in the opinion of the investigator, should preclude use of an investigational agent
- Current skin or soft tissue infection not controlled by antibiotics
- Body weight ≤ 30kg

3 Trial Methods

3.1 Investigational medicinal product

Stage 1

Study Drug	Tocilizumab
Dose Strength	8mg/kg

Dose Regimen	Single Dose
Route of Administration	Infusion over 60 minutes
Duration of Treatment	Day 1

Stage 2

Study Drug	Tocilizumab
Dose Strength	8mg/kg or<br>4mg / kg
Dose Regimen	Single Dose
Route of Administration	Infusion over 60 minutes
Duration of Treatment	Day 1

Tocilizumab (RoActemra), is indicated as either monotherapy or in combination with methotrexate (MTX), for

- the treatment of severe, active and progressive rheumatoid arthritis (RA) in adults not previously treated with MTX.
- the treatment of moderate to severe active RA in adult patients who have either responded inadequately to, or who were intolerant to, previous therapy with one or more disease modifying anti-rheumatic drugs (DMARDs) or tumour necrosis factor (TNF) antagonists.
- the treatment of chimeric antigen receptor (CAR) T cell-induced severe or life-threatening cytokine release syndrome (CRS) in adults and paediatric patients 2 years of age and older.

3.1.1 Method of Administration

After dilution, tocilizumab is administered as an intravenous infusion over 1 hour. In patients ≥ 30 kg tocilizumab should be diluted to a final volume of 100 mL with sterile, non-pyrogenic sodium chloride 9 mg/mL (0.9%) solution for injection using aseptic technique. For instructions on dilution of the medicinal product before administration, see appendix A.

Contraindications

- Known hypersensitivity to tocilizumab
- Active, severe infections
- Known history of diverticular disease
- Active hepatic disease

Monitoring

- Liver enzymes
- Lipid parameters

3.1.2 Special warnings and precautions for use

- The effects of tocilizumab on C-reactive protein (CRP), neutrophils and signs and symptoms of infection should be considered when evaluating patients.

- Tuberculosis: As recommended for other biological treatments, in patients with a history of untreated latent tuberculosis (TB) infection tocilizumab therapy should be avoided.
- Viral reactivation (e.g. hepatitis B virus) has been reported with biologic therapies.
- Events of diverticular perforations as complications of diverticulitis have been reported uncommonly with tocilizumab in RA patients. Tocilizumab should be used with caution in patients with previous history of intestinal ulceration or diverticulitis.
- Hypersensitivity reactions: Serious hypersensitivity reactions have been reported in association with infusion of tocilizumab (see appendix A). Such reactions may be more severe, and potentially fatal in patients who have experienced hypersensitivity reactions during previous infusions even if they have received premedication with steroids and antihistamines. Appropriate treatment should be available for immediate use in the event of an anaphylactic reaction during treatment with RoActemra.
- Transient or intermittent mild and moderate elevations of hepatic transaminases have been reported commonly with RoActemra treatment.

3.1.3 Interactions with other medicinal products

- The expression of hepatic CYP450 enzymes is suppressed by cytokines, such as IL-6, that stimulate chronic inflammation. Thus, CYP450 expression may be reversed when potent cytokine inhibitory therapy, such as tocilizumab, is introduced. *In vitro* studies with cultured human hepatocytes demonstrated that IL-6 caused a reduction in CYP1A2, CYP2C9, CYP2C19 and CYP3A4 enzyme expression. Tocilizumab normalises expression of these enzymes. In a study in RA patients, levels of simvastatin (CYP3A4) were decreased by 57% one week following a single dose of tocilizumab, to the level similar to, or slightly higher than, those observed in healthy subjects.
- When starting or stopping therapy with tocilizumab, patients taking medicinal products which are individually adjusted and are metabolised via CYP450 3A4, 1A2 or 2C9 (e.g. methylprednisolone, dexamethasone, (with the possibility for oral glucocorticoid withdrawal syndrome), atorvastatin, calcium channel blockers, theophylline, warfarin, phenprocoumon, phenytoin, ciclosporin, or benzodiazepines) should be monitored as doses may need to be increased to maintain therapeutic effect. Given its long elimination half-life (t1/2), the effect of tocilizumab on CYP450 enzyme activity may persist for several weeks after stopping therapy.

3.1.4 More common undesirable effects

Infections
Very Common: Upper respiratory tract infections
Common: Cellulitis, Pneumonia, Oral herpes simplex,

Gastrointestinal Disorders
Common:_Abdominal pain, Mouth ulceration, Gastritis

Skin Disorders:
Common:_Rash, Pruritus, Urticaria

Nervous system disorders
Common: Headache, dizziness

Vascular Disorders
Common: Hypertension

Blood Disorders
Common: Leukopenia, Neutropenia, elevated fibrinogen

Eye disorders
Common: Conjunctivitis

A full list of undesirable effects is provided within SPC (see appendix A)

3.1.5 Overdose

There are limited data available on overdose with tocilizumab. One case of accidental overdose was reported in which a patient with multiple myeloma received a single dose of 40 mg/kg. No adverse reactions were observed. No serious adverse reactions were observed in healthy volunteers who received a single dose up to 28 mg/kg, although dose limiting neutropenia was observed

3.2 Supply, Packaging and Storage

Tocilizumab will be supplied by each site hospital pharmacy department.

The medication will be stored in a safe place and in accordance with the conditions defined in the SmPC at the relevant site Hospital Pharmacy Department.

A designated study pharmacist will perform the additional labelling (see appendix B) with study specific information as per Annex 13 GMP requirements. The study pharmacist will also keep detailed records including accountability, labelling and dispensation logs

3.3 Informed Consent:

It is the responsibility of the Investigator to ensure that Subjects meet the eligibility criteria for the study.

The investigator must obtain documented, freely-given consent from each potential subject or each subject's legally acceptable representative prior to any protocol-specific procedures being conducted on that subject and after the subject has read the trial Patient Information Statement (PIS).

An oral explanation should be provided to the subject in addition to the written information and the investigator is responsible for ensuring that the subject understands all the aspects of the study prior to obtaining consent. The investigator may delegate part of this responsibility to the other site staff but should always be involved in the consent process.

Before informed consent may be obtained, the investigator, or a person designated by the investigator, should provide the subject, or the subject's legally acceptable representative, ample time and opportunity to inquire about details of the trial and to decide whether or not to participate in the trial. All questions about the trial should be answered to the satisfaction of the subject or the subject's legally acceptable representative.

Consent must be documented by the subject's dated signature or by the subject's legally acceptable representative's dated signature on a consent form along with the dated signature of the person conducting the consent discussion.

A copy of the PIS and a copy of the signed and dated consent form should be given to the subject or the subject's legally acceptable representative before participation in the trial. However, if this is not possible because of infection control risks associated with potentially contaminated consent forms, this can be deferred until a sufficient period of time has elapsed to ensure that this process is safe to perform. The consent process and most recent PIS and Consent Form version that has been approved by the ethics committee and HRCDC (where applicable) will be followed in any case.

If a subject is unable to read or if a legally acceptable representative is unable to read, an impartial witness should be present during the entire informed consent discussion. After the written informed consent form and any other written information to be provided to subjects, is read and explained to the subject or the subject's legally acceptable representative, and after the subject or the subject's legally acceptable representative has orally consented to the subject's participation in the trial and, if capable of doing so, has signed and personally dated the informed consent form, the witness should sign and personally date the consent form. By signing the consent form, the witness attests that the information in the consent form and any other written information was accurately explained to, and apparently understood by, the subject or the subject's legally acceptable representative, and that informed consent was freely given by the subject or the subject's legally acceptable representative.

In emergency situations, when prior consent of the subject is not possible, the consent of the subject's legally acceptable representative, if present, should be requested. When prior consent of the subject is not possible, and the subject's legally acceptable representative is not available, enrolment of the subject should require measures described in the protocol and/or elsewhere, with documented approval/favourable opinion by the IRB/IEC, to protect the rights, safety and well-being of the subject and to ensure compliance with applicable regulatory requirements. The subject or the subject's legally acceptable representative should be informed about the trial as soon as possible and consent to continue and other consent as appropriate should be requested.

3.4 Inclusion/Exclusion Criteria:

All Subjects must meet all of the inclusion criteria and none of the exclusion criteria to be eligible to participate in this study.

In addition, no treatment or intervention deemed necessary or appropriate will be restricted to study subjects as a result of participating in this trial.

3.5 Inclusion criteria

- Aged 18 years or older
- Confirmed SARS-CoV2 infection (as defined by positive PCR)
- Evidence of hyper inflammatory state as evidenced by at least three of the following:
    - Documented temperature >38°C in the past 48 hours
    - IL6 >40 pg/ml, or in its absence D-dimer >1.5 µgFEU /ml.
    - Elevated CRP (>100mg/L) and/or a three-fold increase since presentation
    - Elevated ferritin X5 ULN
    - Elevated LDH (above the ULN)
    - Elevated fibrinogen (above the ULN)
- Pulmonary infiltrates on chest imaging
- Moderate to severe respiratory failure as defined by $PaO_2/FiO_2 \leq 300$mmHg

3.6 Exclusion criteria

- Primary or secondary immunodeficiency
- Use of significant immunosuppressive therapy in the last 3 months (not including hydroxychloroquine or short course of corticosteroids (defined as <400mg cumulative dose)
- Active malignancy requiring treatment

- Known active current or history of recurrent bacterial, mycobacterial, fungal or viral infections including history of untreated latent TB
- History of diverticulitis or chronic ulcerative GI disease that might predispose to GI perforation
- Severe allergic reaction to monoclonal antibodies
- Pregnancy or breast feeding
- AST / ALT with values greater than 10 times normal levels or history of significant liver disease that in the opinion of the investigator precludes use of an investigational agent
- Neutrophils < 0.5 x10^9/L
- Platelets < 50x10^9/L
- Documented, uncontrolled sepsis caused by other pathogen(s) other than COVID-19
- Presence of co-morbidities (including cognitive impairment and/or frailty) that, in the opinion of the investigator, should preclude use of an investigational agent
- Current skin or soft tissue infection not controlled by antibiotics
- Body weight ≤ 30kg

3.7 Clinical and medication history

3.7.1 Demographics:

Subjects' demographic data will be collected. The data required from all subjects include; sex, age, ethnic group, country of birth, race, social living conditions, smoking (including vaping), highest attained educational status and alcohol consumption.

3.7.2 COVID-19 History:

A detailed history will be obtained from the subject on transmission source of COVID-19, symptoms experienced and date and time of onset of symptoms as well as first confirmation of COVID-19 diagnosis by PCR. In addition, where available, information on pre-existing abnormalities on chest x-ray, predating onset of COVID-19 symptoms, will be recorded.

3.7.3 Medical History:

Medical history including date of onset of any symptoms, date of onset of fever (if known), date of first reported breathlessness and date of confirmed COVID-19 diagnosis will be recorded at screening visit in the source notes.

3.7.4 Concomitant medications:

Clinically significant drug-drug interactions may occur with the medicinal products used to treat COVID-19. A thorough medication history (including alternative and herbal medicines) should be obtained prior to initiation of treatment.

All medications including prescription medication, over the counter medications and herbal remedies to be recorded. Start dates, dose and frequency of each medication to be included where available.

Throughout the trial, all new and current concomitant medications (including oxygen therapy) with start/ stop dates will be recorded.

3.8 Screening Assessments / Procedures

3.8.1 Physical Examination

A complete physical examination (including measurement of height (cms) and weight (kg) from which body mass index will be calculated (kg/m^2)) will be performed at screening and a symptom-directed physical examination will be performed at follow up visits. Information from physical examination must be documented in the source notes. Height and weight will be recorded (self -reported height and weight can be used if unable to complete at screening visit.)

Clinically significant findings must be reported as medical history or an adverse event.

3.8.2 Vital signs

Vital signs will be collected at each study timepoint; if vital signs are routinely recorded by hospital staff, the most recent recording and the highest temperature in the preceding 24 hours can be used for the purposes of the study. Date and time of vital sign measurements should be recorded. Vital signs include systolic and diastolic blood pressure, heart rate, respirations, pulse oximetry oxygen saturations (include fraction of inspired oxygen (FiO_2) and route of oxygen therapy administration if applicable) and temperature.

3.8.3 ECG

A 12 lead ECG will be obtained at screening unless an ECGs recorded within 36 hours prior to screening is available, in which case it can be used as a replacement for this screening ECG. Interpretation of the ECG tracing must be made by a qualified doctor. Clinically significant findings must be reported as medical history or an adverse event.

3.8.4 Chest X-ray:

A chest x-ray will be obtained at screening unless a chest x-ray has been performed within 36 hours prior to screening, in which case it can be used as a replacement for the screening chest x-ray. Repeat chest x-rays will be performed at days 2, 4 and 6. Additional chest x-rays may be requested as part of the study at days 14 and 28 if residual changes related to COVID19 infection persist. Clinically significant chest x-ray findings must be reported as medical history or an adverse event.

3.8.5 Pregnancy test:

Female subjects of childbearing potential must have a negative serum or urine pregnancy test (βHCG) to be eligible to participate in this study. If a urine pregnancy test is positive, it must be confirmed with a serum pregnancy test.

A woman is considered of childbearing potential (WOCBP), i.e. fertile, following menarche and until becoming post-menopausal unless permanently sterile. Permanent sterilisation methods include hysterectomy, bilateral salpingectomy and bilateral oophorectomy.

3.8.6 Adverse events:

Adverse events and serious adverse events will be recorded as per schedule of events. The Investigator is responsible for assessing adverse events and serious adverse events for causality and severity. Refer to Safety Reporting Requirements/Data and Safety Monitoring Plan.

3.9 Assignment of Study Number

All eligible subjects will be assigned a unique study number at screening. The study number identifies the subject for all procedures occurring after enrolment.

3.10 Randomisation

Eligible patients will be randomized (1:1) using a central register. Randomisation will be performed through an interactive, web-based electronic data capturing database.

In stage 1, eligible participants will randomised (1:1) to standard of care with single dose (8mg/kg, maximum 800mg) intravenous tocilizumab infused over 60 minutes or to standard of care alone.

Stage 1.
- Arm 1. Standard of care
- Arm 2. Standard of care plus single dose (8mg/kg, maximum 800mg) intravenous tocilizumab infused over 60 minutes.

In stage 2, eligible participants will be randomised (1:1) to receive either single, standard dose (8mg/kg, maximum 800mg) intravenous tocilizumab infused over 60 minutes or reduced dose (4mg/kg, maximum 800mg) intravenous tocilizumab infused over 60 minutes.

Stage 2.
- Arm 1. Standard of care plus single, standard dose (8mg/kg, maximum 800mg) intravenous tocilizumab infused over 60 minutes
- Arm 2. Standard of care plus single, reduced dose (4mg/kg, maximum 800mg) intravenous tocilizumab infused over 60 minutes

The randomization number identifies the subject alongside the study screening number for all procedures occurring after randomisation. Once a randomization number is assigned to a subject, it can never be reassigned to another subject.

3.11 Clinical assessments / Procedures

Following informed consent and screening for eligibility (including reviews of available chest x-rays), participants will be recruited only to stage 1. Once stage 1 has fully recruited, subsequent participants will be enrolled directly to stage 2.

All clinical assessments will be conducted according to the Schedule of Visits.

3.11.1 Screening:

After providing written, informed consent, subjects will undergo the following assessments:

- Subjects will be asked to provide demographics, medication and clinical history
- Assessments of current symptoms
- Full physical examination (including height, weight and calculation of BMI)
- Recording of vital signs
- ECG (if not performed in the previous 36 hours)
- Chest x-ray (if not performed in the previous 36 hours)
- Clinical chemistry and haematology (if not performed in the previous 24 hours)
- Lipid profile – total cholesterol, HDL cholesterol, LDL cholesterol and triglycerides (fasting or non-fasting)
- HbA1C
- Inflammatory markers (ferritin, fibrinogen, D-dimer, LDH, CRP and/or IL6 (if available) if not performed in the previous 24 hours
- Pregnancy test, in women of childbearing potential (serum/urine βHCG)

- Swab for SARS CoV-2 PCR (if previous result not available from the current inpatient admission)

Once the screening visit is complete if the subject is eligible for the study, the subject can proceed immediately to the baseline visit on the same day.

3.11.2 Baseline visit (Day 0):

The following assessments will be conducted:

- Randomisation as per stage of study (stage 1 or stage 2)
- Assessments of current symptoms
- Symptom directed physical examination
- Recording of vital signs
- Clinical chemistry and haematology (only if screening visit and baseline visit not performed on the same day)
- Inflammatory markers (ferritin, fibrinogen, troponin, LDH, CRP, D-dimer and/or IL6 (if available)) only if screening visit and baseline visit not performed on the same day
- HIV serology
- Hepatitis B serology (s-antigen, s-antibody and c-antibody)
- Quantiferon
- Aerial blood gas (ABG) (if clinically indicated)
- Chest x-ray (if not available within the previous 36 hours)
- Sample storage (as per appendix C)
    - Spot urine sample in sterile container (no preservative)
    - Respiratory samples (nasopharyngeal swabs and/or sputum)
    - Stool (first available sample post randomisation)
    - Blood samples for storage

If randomised to an active drug arm, participants will be administered tocilizumab at the dose allocated through randomisation.

3.11.3 Day 1 visit:

The following assessments will be conducted:

- Assessments of current symptoms
- Symptom directed physical examination
- Recording of vital signs
- Clinical chemistry and haematology
- Inflammatory markers (ferritin, fibrinogen, troponin, LDH, CRP, D-dimer and/or IL6 (if available))
- Aerial blood gas (ABG) (if clinically indicated)

If randomised to an active drug arm, participants will be administered tocilizumab at the dose allocated through randomisation.

3.11.4 Day 2 visit:

The following assessments will be conducted:

- Assessments of current symptoms
- Symptom directed physical examination
- Recording of vital signs
- Clinical chemistry and haematology
- Inflammatory markers (ferritin, fibrinogen, troponin, LDH, CRP, D-dimer and/or IL6 (if available))
- Aerial blood gas (ABG) (if clinically indicated)
- Chest x-ray (if not available within the previous 36 hours)
- Sample storage (as per appendix C)
  - Respiratory samples (nasopharyngeal swabs and/or sputum)
  - Blood samples for storage

If randomised to an active drug arm, participants will be administered tocilizumab at the dose allocated through randomisation.

3.11.5 Day 4 visit:

The following assessments will be conducted:

- Assessments of current symptoms
- Symptom directed physical examination
- Recording of vital signs
- Clinical chemistry and haematology
- Inflammatory markers (ferritin, fibrinogen, troponin, LDH, CRP, D-dimer and/or IL6 (if available))
- Aerial blood gas (ABG) (if clinically indicated)
- Chest x-ray (if not available within the previous 36 hours)

3.11.6 Day 8 visit

The following assessments will be conducted:

- Assessments of current symptoms
- Symptom directed physical examination
- Recording of vital signs
- Clinical chemistry and haematology
- Lipid profile – total cholesterol, HDL cholesterol, LDL cholesterol and triglycerides (fasting or non-fasting)
- Inflammatory markers (ferritin, fibrinogen, troponin, LDH, CRP, D-dimer and/or IL6 (if available))
- Aerial blood gas (ABG) (if clinically indicated)
- Chest x-ray (if not available within the previous 36 hours)
- Sample storage (as per appendix C)

  - Spot urine sample in sterile container (no preservative)
  - Respiratory samples (nasopharyngeal swabs and/or sputum)
  - Stool (first available sample post randomisation)
  - Blood samples for storage

3.11.7 Day 14 visit

The following assessments will be conducted:

- Assessments of current symptoms
- Symptom directed physical examination
- Recording of vital signs
- Clinical chemistry and haematology (if clinically indicated)
- Inflammatory markers (ferritin, fibrinogen, troponin, LDH, CRP, D-dimer and/or IL6 (if available))
- Aerial blood gas (ABG) (if clinically indicated)
- Chest x-ray (if residual changes persist on previous chest x-ray)
- Sample storage (as per appendix C)
  - Spot urine sample in sterile container (no preservative)
  - Respiratory samples (nasopharyngeal swabs and/or sputum)
  - Stool (first available sample post randomisation)
  - Blood samples for storage

3.11.8 Day 28 visit

The following assessments will be conducted:

- Assessments of current symptoms
- Symptom directed physical examination
- Recording of vital signs
- Clinical chemistry and haematology (if clinically indicated)
- Inflammatory markers (ferritin, fibrinogen, troponin, LDH, CRP, D-dimer and/or IL6 (if available))
- Chest x-ray (if residual changes persist on previous chest x-ray)
- Sample storage (as per appendix C)
  - Spot urine sample in sterile container (no preservative)
  - Respiratory samples (nasopharyngeal swabs and/or sputum)
  - Stool (first available sample post randomisation)
  - Blood samples for storage

3.12 Laboratory assessments

3.12.1 Laboratory Assessments:

Laboratory assessments will be carried out at the trial site's local laboratory. The timing for these laboratory procedures/assessments are outlined in the Schedule of Visits.

The following laboratory samples will be collected as per schedule of assessments:

- Blood tests (for reference ranges see appendix D)

  - Haematology
  - Chemistry panel (renal, liver (including AST and ALT), bone panel and CK)
  - Lipid profile
  - HbA1C
  - CRP
  - LDH
  - Ferritin
  - IL-6
  - Troponin
  - Fibrinogen
  - D-dimer
  - Arterial blood gas (if clinically indicated)
  - Pregnancy testing (blood or urine)
- Nasopharyngeal swab or sputum for SARS-CoV-2 PCR testing
- Sample storage (*see appendix C*)

3.12.2 Stored samples

Blood, urine, stool and respiratory samples will be stored to enable future studies.

Blood samples for serum, plasma and PBMC storage as well as respiratory samples will be taken at day 0, 2, 8, 14 and 28. In addition, stool and urine samples will be collected ay day 0, 8, 14 and 28 (*see schedule of visits*).

These samples will be processed centrally at the designated study laboratory by experienced laboratory staff according to a standardised protocol.

The methods for collection and processing laboratory samples prior to analysis, shipping or storage are detailed in the study Laboratory Manual. It is the responsibility for the investigator at each site to ensure that all site staff who will be handling, packaging, and/or shipping biological samples understand and comply with International Air Transport Association (IATA) regulations relating to the handling and shipping of hazardous goods and/or diagnostic specimens.

All stored samples will be identified by a study code, designation of study visit and date of collection and data linkage to the subject's medical record number (MRN) will be contained on a password-protected computer file located on a secure research computer network.

Samples will be stored at -80ºC in a dedicated, monitored sample storage facility within the designated study laboratory. The storage facility should includes 24-hour real-time remote monitoring of freezers with alarms to ensure integrity of samples for the duration of the study.

4 Assessment of safety

Timely, accurate and complete reporting and analysis of safety information from clinical studies are crucial for the protection of patients and are mandated by regulatory agencies worldwide.

Data from all subjects entering the study will be included in the analysis of safety. The number of adverse events (AEs) and serious adverse events (SAEs) will be tabulated by severity and treatment received. All such events will be recorded on the adverse event case report forms. *(See appendix G -Adverse Events and Serious Adverse Events definition*).

4.1 Evaluation of AEs and SAEs

Seriousness, causality, severity and expectedness should be evaluated.

4.1.1 Assessment of seriousness

A Serious Adverse Event (SAE) is defined as any untoward medical occurrence or affect that at any dose:

- results in death,
- is life-threatening*,
- requires hospitalisation or prolongation of existing hospitalisation,
- results in persistent or significant disability or incapacity,
- is a congenital anomaly or birth defect
- important medical events**

*Regarding a life-threatening event, this refers to an event in which the subject was at risk of death at the time of the event; it does not refer to an event which hypothetically might have caused death if it were more severe.

**Some medical events may jeopardise the subject or may require an intervention to prevent one of the above characteristics/consequences. Such events (hereinafter referred to as 'important medical events') should also be considered as 'serious' in accordance with the definition

The investigator should make an assessment of seriousness as defined.

- AE results in death
- AE is life-threatening

Medical and scientific judgement should be exercised in deciding whether expedited reporting is appropriate in other situations, such as important medical events that may not be immediately threatening or result in death or hospitalisation, but may jeopardise the subject or may require intervention to prevent one of the outcomes listed in the SAEs definition.

4.1.2 Assessment of causality

The investigator's assessment of causality must be made for all AEs (serious and non-serious).

All adverse events reported by study subjects will be captured and reported. Additional data will also be captured on the relationship between a reported AE and the IMP as per the judgement of the investigator and / or the sponsor as having a reasonable suspected causal relationship to an investigational medicinal product.

The causality assessment given by the investigator should not be downgraded by the sponsor.

The investigator must make an assessment of whether the AE/SAE is likely to be related to treatment according to the following definitions:

- **Unrelated:** Where an event is not considered to be related to the study medication.
- **Possibly:** Although a relationship to the study medication cannot be completely ruled out, the nature of the event, the underlying disease, concomitant medication or temporal relationship make other explanations possible.
- **Probably:** The temporal relationship and absence of a more likely explanation suggest the event could be related to the study medication.

All AEs/SAEs judged as having a reasonable suspected causal relationship (e.g. possibly, probably) to the study medication will be considered as adverse reactions (ARs) or serious adverse reactions (SARs) – *see appendix G.* All AEs/SAEs judged as being related (e.g. possibly, probably) to an interaction between the study medication and another medication will also be considered to be ARs/SAR.

Alternative causes such as natural history of the underlying disease, concomitant therapy, other risk factors and the temporal relationship of the event to the treatment should be considered.

4.1.3 Assessment of severity

The investigator will make an assessment of severity for each AE/SAE and record this on the CRF according to one of the following categories:

- **Mild:** An event that is easily tolerated by the subject, causes minimal discomfort and does not interfere with everyday activities.
- **Moderate:** An event that is sufficiently uncomfortable to interfere with normal everyday activities.
- **Severe:** An event that prevents normal everyday activities.

4.1.4 Assessment of expectedness

The expectedness of an adverse reaction will be determined by the sponsor according to the reference document. For the study drugs involved in this study which have a marketing authorization (commercial agent), the expectedness of an adverse event will be determined by whether or not it is listed in the summary of product characteristics (SmPC).

For this study, the current version of the tocilizumab SPC will be used to assess the expectedness of the event in the study (*See Appendix A*)

4.1.5 Relation to Study IMP

The investigator (or designee) must determine the likelihood that the IMP caused the adverse event. The investigator must record the causal relationship in the patients' notes, as appropriate, and report such an assessment in accordance with serious adverse reporting requirements, if applicable.

An adverse event is considered associated with the use of the IMP/treatment if the attribution is definitely, probably or possibly related.

Procedures for Recording and Reporting Adverse Events

The site clinical investigator is responsible for the detection and documentation of events meeting the definition of AE or SAE as provided in this protocol.

Only non-serious Adverse Events which are considered related to the IMP will be recorded routinely on the Adverse Event eCRF from the time of informed consent (or enrolment into the study) up to 90 days after the last dose of study drug has been received. All Adverse Events meeting "serious" criteria should be recorded on the Adverse Event eCRF as per Section below.

4.2 Procedures for Recording and Reporting Serious Adverse Events

AEs meeting the definition of SAEs must be reported using the SAE Report Form located in the Investigator Site File (ISF). All AEs meeting "serious" criteria occurring in each patient should be reported from the time of informed consent (or enrolment into the study) up to 90 days after the last dose of study drug has been received.

The Investigator will submit to the UCD Pharmacovigilance unit, using the SAE report forms provided, all Serious Adverse Events in the study regardless of whether causality with the administration of study drug is suspected by the investigator. The Investigator will transmit the SAE reports by email to the pharmacovigilance email immediately i.e. defined as within 24 hours of a member of the research team becoming aware of the event(s).

Any SAEs occurring in patients after 90 days of the last dose of study drug, which are deemed by the investigator to be causally related to study drug should be forwarded to UCD Pharmacovigilance Unit as outlined above.

All SAE reports to be emailed immediately to: sae.reporting@ucd.ie

UCD Pharmacovigilance will acknowledge receipt of the SAE via email to the reporter and/or Investigator.

All SAEs should be monitored until they are resolved or are clearly determined to be due to a patient's stable or chronic condition or intercurrent illness(es). Information not available at the time of the initial report e.g. pending lab results must be documented on a follow-up SAE report form. Follow-up information should be sought and submitted as it becomes available. The follow-up information should describe whether the event has resolved or persists, if and how it was treated and whether the patient continues on study or has been withdrawn from treatment.

If unsure whether an event meets seriousness criteria, please contact the Pharmacovigilance unit at the above email address who will deal with your query and advise on specific reporting requirements.

4.3 Sponsor Responsibilities:

The Sponsor, UCD, will perform appropriate adverse event reporting for the study according to the applicable regulatory guidelines. UCD will submit local SUSARs on an expedited basis to the local regulatory authority and other Competent Authorities involved, concerned Ethics Committees (EC) and all participating investigators. UCD sends annual Development Safety Update Reports (DSUR) to the concerned regulatory authority and Ethics Committee. Investigators will be informed of SUSARs.

4.4 Procedures for Documenting and Reporting Pregnancies

Where a female subject is enrolled and becomes pregnant during the course of the study must be reported to UCD Pharmacovigilance unit by the investigator within 24 hours of the study site becoming aware of the event, by email using the Pregnancy Report Form located in the Investigator Site File (ISF). Forms should be sent to the email address sae.reporting@ucd.ie.

The Pharmacovigilance unit will acknowledge receipt of this form by email. Any patient who becomes pregnant during the study must be promptly withdrawn from the study following withdrawal procedures outlined in protocol section '*Study Procedures – subject withdrawal criteria and procedures for withdrawal*'. The pregnancy will be monitored closely until the birth. Follow-up information regarding the outcome of the pregnancy and any postnatal sequelae in the infant will be required.

5 Statistical plan and Interim analysis

Stage 1 analysis:

Analyses of efficacy endpoints will be done on the intention-to-treat population, and safety in all patients who received at least one dose of single dose of intravenous tocilizumab or standard of care. Categorical will be summarized using counts and proportions and continuous data using mean (standard deviation) or median (interquartile range), as appropriate. The composite primary-endpoint (time to intubation and ventilation, non-invasive ventilation or death) will be estimated using Kaplan-Meier methods and compared between treatment groups with the log-rank test. Hazard ratios for the composite primary endpoint and 95% confidence intervals (CIs) comparing the treatment arms will be estimated using Cox proportional hazards models. Exploratory analyses on change in secondary continuous end-points from baseline to 28 days after randomisation will be compared between the two treatments groups by analysis of covariance (ANCOVA) adjusted for baseline values. Where appropriate, continuous

outcomes endpoints may be log-transformed prior to analysis. Time to event secondary endpoints will be explored using a similar approach to the primary endpoint.

Adverse event (AE) term recorded during the study will be mapped to a system organ class and preferred term using the current MedDRA Dictionary (Version 10.0) or later compared using the chi-square tests. A post-hoc procedure to account for multiplicity in tests of secondary end-points, with a Bonferroni-adjusted significance level will applied to any claim for benefit in the secondary end points.

Stage 2 analysis:

Sample size calculation for this dose evaluation stage will use data generated using the same primary endpoint as in Stage 1.

To establish efficacy, differences in composite endpoint event rates between arms will be evaluated using the following criteria propose by Sin-Ho Jung [15]. Briefly using a sample size ($(n, n1, a1, a)$ calculated after Phase 1, we will randomised n1 patients to each arm. Let X1 and Y1 denote the number with composite endpoint events among the n1 first stage patients randomised to standard of care + 8mg/kg (A) and standard of care + 4mg/kg (B) intravenous tocilizumab, respectively. We proceed to the second stage if $X1 - Y1 \geq a1$ for a chosen integer $a1 \in [-n1, n1]$. Otherwise, we reject treatment A and stop the trial. In the second stage, we accrue additional n2 patients to each arm. Let X2 and Y2 denote the number with composite endpoint events among the second stage patients of treatment A and B, respectively. Also, letting $X = X1 + X2$ and $Y = Y1 + Y2$ denote the total number with composite endpoint events among the cumulative $n = n1 + n2$ patients for treatment A and B2, respectively. For an integer $a \in [a1 - n2, n]$, we accept treatment A for further investigation if $X - Y \geq a$. Otherwise, we reject treatment A.

Exploratory evaluation of the primary and secondary endpoints will be similar to those used in the analysis in Phase 1 of the study.

6 DATA MANAGEMENT

The site investigator will facilitate the permit of study-related monitoring, audit, research ethics committee approval review and regulatory inspection by the relevant national authority, providing direct access to source documents were necessary.

Data will be generated and managed according to the study specific data management plan set out in the study site file. Sources of data will be identified and pre-specified on a study-specific source data agreement relevant to the study site. The source data agreement will be verified by the site Investigator with a copy of the agreement to be filed within the Site File.

The study data will be collected using Case Report Forms (CRFs). A case report form (CRF) is designed to record the data required by the protocol and collected by the investigator in the patient’s clinical source documents.

Source documents for this study will include hospital records, procedure reports and data collection forms. Any data to be entered directly on the Case Report Forms will be considered to be source data. Source documents will be stored in a secure location as per hospital/site policy.

Data collected will be coded. On all study-specific documents other than the signed consent, the subject will be referred to by the study subject identification number/code. Any reports that acts as a source document, other than completed CRF, should be signed and dated by a listed site investigator for issues of medical significance (for example the review of laboratory reports).

Subject participation and subject progress should also be recorded in the subject medical records to ensure relevant healthcare providers have knowledge of the subject's participation in the study.

Clinical trial data will be entered by authorised site personnel into the electronic data capture system. The data should be verifiable against original records and source notes by the Monitor during monitoring visits. Data reported on the eCRF that are derived from source documents must be consistent with the source documents or the discrepancies must be explained.

CRF completion instructions will be provided and any questions about recording specific information on the CRF should be directed to the Monitor.

6.1 Data Quality Assurance

All data will be reviewed for completeness and logical consistency by the Data Management team. Data queries will be generated via the electronic data capture system to correct or clarify data or request missing information. The designated site staff will be required to respond to these queries in accordance with data entry and data query timelines for the study.

The investigator will be responsible for the review and sign off of data entered and corrected for their site.

6.2 Record Retention

An investigator site file will be provided by UCD for all required study documents. The investigator will maintain all study records according to ICH-GCP and applicable regulatory requirement(s). Essential documents will be retained until at least 15 years after the publication of the clinical study report. These documents should be retained for a longer period however if required by the applicable regulatory requirements or by an agreement with the sponsor.

The investigator/institution should agree to retain the trial-related essential documents as required by the applicable regulatory requirements and until the sponsor informs the investigator/institution these documents are no longer necessary.

6.3 Confidentiality of Subject Records

By signing the protocol, the investigator agrees that the sponsor, ethics committee or regulatory authorities may consult and/or copy study documents to verify information in the case record form. By signing the consent form the subject agrees to this process.

Subject confidentiality will be maintained at all times and no documents containing the subject's name or other identifying information will be collected by the sponsor on the study database. It may be necessary for the sponsor's representatives, the ethics committee and regulatory authority representatives to have direct access to the subject's medical records. If study documents need to be photocopied during the process of verifying case record form data, the subject will be identified by a unique code only; full names and other identifying information will be masked.

6.4 Confidentiality of Study Data

By signing this protocol, the investigator affirms to the sponsor that information provided to the investigator by the sponsor will be maintained in confidence and will be divulged only as necessary to the ethics committee and institution employees directly involved in the study. Both ethics committee members and employees must also understand the confidentiality requirements for any information divulged to them. The data generated by

this study will be considered confidential by the investigator, except to the extent that it is included in a publication as agreed in the publication policy of this protocol.

7 DATA AND SAFETY MONITORING BOARD

An independent Data and Safety Monitoring Board (DSMB) will be established to perform ongoing safety surveillance and to perform interim analyses on the study data.

The DSMB will be an independent committee, composed of a minimum of three members; at least two will be clinicians not involved in the trial but with experience and expertise in clinical trials and / or biostatistics; at least one member will be a clinician with expertise in infectious diseases. Each member will state that they have no conflict of interest with the sponsor involved in the study or any other conflicting interest to declare. The DSMB will be supported by the study statistician.

The DSMB will meet on three pre-defined occasions. The first meeting will be convened when 5 subjects who have received IMP have completed day 8 of study to decide if the safety of the planned intervention is acceptable. The second meeting will be convened at the pre-planned interim analysis once 16 endpoints have been accrued in stage 1 and the third meeting will be convened once stage 1 has been fully recruited and all participants have reached day 8. At meeting one, the DSMB will be provided with relevant updated analyses of safety and at meetings two and three, with analyses of both safety and efficacy by the study statistician. Any imbalances in prevalence of and/or severity of adverse events between arms will be addressed. Based on the data provided, the DSMB may make one of the following recommendations to the sponsor:

1. Discontinue the clinical trial or stage of the clinical trial immediately due to concerns around safety and / or lack of efficacy
2. Discontinue one of the study arms where there are concerns around safety
3. Continue with the clinical trial

The DSMB will consider findings from any other relevant studies and review trial data on recruitment, safety, and adherence to trial strategies and efficacy in strict confidence. Where new findings are published that may impact on the safe conduct of the trial, the sponsor may request additional DSMB meetings to consider such evidence. If the DSMB observe any significant excess of serious adverse events in any treatment group, they may recommend premature termination of the trial on the basis of serious safety concerns. The group will consider the progress of the study and make recommendations to the sponsor where necessary.

8 REGULATORY APPROVAL

Regulatory approval from the Health Products Regulatory Authority (HPRA) will be obtained prior to conduct of the clinical trial in Ireland. This study will be conducted in accordance with the regulatory requirements, sponsor standard operating procedures (SOPs), ICH GCP and ethical principles laid out in the Declaration of Helsinki (2013) and the National Statement on Ethical Conduct in Research involving Humans *(See Appendix H)*.

The investigator is responsible for obtaining ethics committee approval of the protocol in compliance with the local regulatory requirements prior entering any subject into the clinical trial. The approval must clearly identify the protocol and all documents approved

by the ethics committee including version numbers of the protocol and informed consent. A copy of the approval should be sent to the sponsor of the study.

The investigator should also obtain approval for any amendments to the protocol or informed consent during the course of the study. The investigator must comply with all ethics committee reporting requirements for serious adverse events, annual updates and end of study reports and must agree to abide by any ethics committee conditions of approval. The Ethics Committee must review and approve the initial and any amended consent forms prior to use in the study. The subject or the legally acceptable representative should be informed in a timely manner of any new information that becomes available during the course of the study that may affect the subject's willingness to continue participation in the trial. The communication of this information may be provided and documented via a patient letter or revised consent form or addendum to the original consent form that captures the subject's dated signature or by the subject's legally acceptable representative's dated signature.

Specifics about a trial and the trial population will be added to the consent form template at the protocol level.

The informed consent will adhere to IRB/ERC requirements, applicable laws and regulations and Sponsor requirements.

9 QUALITY CONTROL & QUALITY ASSURANCE PROCEDURES

The sponsor will conduct regular monitoring visits throughout the course of the clinical trial, in accordance with ICH GCP. The investigator will allow the monitor to visit the site and facilities where the study will take place in order to ensure compliance with the protocol requirements and ICH GCP. Training sessions may be organised for the investigators and/or instruction manuals may be given to the study team as required.

The Investigator will permit the Sponsor, authorised agents of the Sponsor (such as Study Monitors, auditors, etc.) and regulatory agency employees to enter and inspect any site where the drug or records pertaining to the drug are held, and to inspect and copy all records relating to an investigation, including research participant records.

To ensure the accuracy of data submitted, it is mandatory that representatives of the Sponsor and of the regulatory agencies have access to source documents (i.e. participant medical records or notes, charts, laboratory reports, etc.). Participant confidentiality will be protected at all times. The investigator will allow the monitor to:

- inspect the site, the facilities and the material used for the study,
- meet all members of his/her team involved in the study,
- consult all of the documents relevant to the study,
- have access to the electronic case report forms (i.e. access to an analogic phone line or his/her computer)
- check that the electronic case report forms have been filled out correctly,
- directly access source documents for comparison of data therein with the data in the electronic case report forms,
- verify that the study is carried out in compliance with the protocol and local regulatory requirements.

All data will be stored securely. Details of outcome measures and adverse events will be documented in hospital healthcare records, in individual research participant case report forms and in an encrypted electronic database.

The study investigators will adhere to hospital protocols pertaining to healthcare record use and storage. The investigators and authorised designees will ensure that the confidentiality of the participants' data is preserved.

This trial may be subject to internal or external auditing or inspections procedure to ensure adherence to GCP. Access to all trial-related documents will be given at that time.

A quality assurance audit may be conducted by the sponsor or its agent at any time during, or shortly after, the study. The investigator will permit an independent audit by an auditor mandated by Sponsor, after reasonable notice. The purpose of an audit is to confirm that the study is conducted as per protocol, GCP and applicable regulatory requirements, that the rights and well-being of the patients enrolled have been protected, and that the data relevant for the evaluation of the investigational medicinal product have been captured, processed and reported in compliance with the planned arrangements. The investigator will permit direct access to all study documents, drug accountability records, medical records and source data.

Regulatory authorities may perform an inspection of the study up to several years after its completion. If an inspection is announced the Sponsor will be informed immediately.

10 FINANCING & INSURANCE/INDEMNITY

The UNIVERSITY COLLEGE OF DUBLIN is the SPONSOR and it will ensure that every investigator is covered by a Public Liability ('negligent harm') insurance that applies for the clinical trial. All investigators are qualified and practicing physicians and are thus insured by the clinical indemnity scheme (CIS).

University College Dublin is funding this trial.

All relevant insurance documentation will be obtained prior to conduct of the trial.

11 Bibliography

1. Rubbert-Roth, A., et al., *A Review of Recent Advances Using Tocilizumab in the Treatment of Rheumatic Diseases.* Rheumatol Ther, 2018. **5**(1): p. 21-42.
2. Smolen, J.S., et al., *EULAR recommendations for the management of rheumatoid arthritis with synthetic and biological disease-modifying antirheumatic drugs: 2016 update.* Ann Rheum Dis, 2017. **76**(6): p. 960-977.
3. Hellmich, B., et al., *2018 Update of the EULAR recommendations for the management of large vessel vasculitis.* Ann Rheum Dis, 2020. **79**(1): p. 19-30.
4. Hutchinson, M., R.S. Tattersall, and J.J. Manson, *Haemophagocytic lymphohisticytosis-an underrecognized hyperinflammatory syndrome.* Rheumatology (Oxford), 2019. **58**(Suppl 6): p. vi23-vi30.
5. Watanabe, E., et al., *Successful Tocilizumab Therapy for Macrophage Activation Syndrome Associated with Adult-Onset Still's Disease: A Case-Based Review.* Case Rep Med, 2016. **2016**: p. 5656320.
6. Chen, H., et al., *Management of cytokine release syndrome related to CAR-T cell therapy.* Front Med, 2019. **13**(5): p. 610-617.
7. Le, R.Q., et al., *FDA Approval Summary: Tocilizumab for Treatment of Chimeric Antigen Receptor T Cell-Induced Severe or Life-Threatening Cytokine Release Syndrome.* Oncologist, 2018. **23**(8): p. 943-947.
8. Huang, C., et al., *Clinical features of patients infected with 2019 novel coronavirus in Wuhan, China.* Lancet, 2020. **395**(10223): p. 497-506.
9. Ruan, Q., et al., *Clinical predictors of mortality due to COVID-19 based on an analysis of data of 150 patients from Wuhan, China.* Intensive Care Med, 2020: p. 1-3.
10. Zhou, F., et al., *Clinical course and risk factors for mortality of adult inpatients with COVID-19 in Wuhan, China: a retrospective cohort study.* Lancet, 2020. **395**(10229): p. 1054-1062.
11. Xu, X., Han, Mingfeng,Li, Tiantian,Sun, Wei,Wang, Dongsheng,Fu, Binqing,Zhou, Yonggang,Zheng, Xiaohu,Yang, Yun,Li, Xiuyong,Zhang, Xiaohua,Pan, Aijun,Wei, Haiming, *Effective Treatment of Severe COVID-19 Patients with Tocilizumab.* ([ChinaXiv:202003.00026] 2020.
12. Burmester, G.R., et al., *A randomised, double-blind, parallel-group study of the safety and efficacy of subcutaneous tocilizumab versus intravenous tocilizumab in combination with traditional disease-modifying antirheumatic drugs in patients with moderate to severe rheumatoid arthritis (SUMMACTA study).* Ann Rheum Dis, 2014. **73**(1): p. 69-74.
13. Guan, W.J., et al., *Clinical Characteristics of Coronavirus Disease 2019 in China.* N Engl J Med, 2020.
14. Lachin, J.M. and M.A. Foulkes, *Evaluation of sample size and power for analyses of survival with allowance for nonuniform patient entry, losses to follow-up, noncompliance, and stratification.* Biometrics, 1986. **42**(3): p. 507-19.
15. Jung, S.H., *Randomized phase II trials with a prospective control.* Stat Med, 2008. **27**(4): p. 568-83.

12 Appendices

Appendix A – Tocilizumab Summary of Product Characteristics – SmPC

13 SUMMARY OF PRODUCT CHARACTERISTICS

1. NAME OF THE MEDICINAL PRODUCT

RoActemra 20 mg/mL concentrate for solution for infusion.

2. QUALITATIVE AND QUANTITATIVE COMPOSITION

Each mL concentrate contains 20 mg tocilizumab*.

Each vial contains 80 mg of tocilizumab* in 4 mL (20 mg/mL). Each vial contains 200 mg of tocilizumab* in 10 mL (20 mg/mL). Each vial contains 400 mg of tocilizumab* in 20 mL (20 mg/mL).

***humanised IgG1 monoclonal antibody against the human interleukin-6 (IL-6) receptor produced in Chinese hamster ovary (CHO) cells by recombinant DNA technology.**

Excipients with known effects

Each 80 mg vial contains 0.10 mmol (2.21 mg) sodium. Each 200 mg vial contains 0.20 mmol (4.43 mg) sodium. Each 400 mg vial contains 0.39 mmol (8.85 mg) sodium.

For the full list of excipients, see section 6.1.

3. PHARMACEUTICAL FORM

Concentrate for solution for infusion

(sterile concentrate). Clear to opalescent,

colourless to pale yellow solution.

4. CLINICAL PARTICULARS

4.1 Therapeutic indications

RoActemra, in combination with methotrexate (MTX), is indicated for:

- the treatment of severe, active and progressive rheumatoid arthritis (RA) in adults not previously treated with MTX.
- the treatment of moderate to severe active RA in adult patients who have either responded inadequately to, or who were intolerant to, previous therapy with one or more disease- modifying anti-rheumatic drugs (DMARDs) or tumour necrosis factor (TNF) antagonists.

In these patients, RoActemra can be given as monotherapy in case of intolerance to MTX or where continued treatment with MTX is inappropriate.

RoActemra has been shown to reduce the rate of progression of joint damage as measured by X-ray and to improve physical function when given in combination with methotrexate.

RoActemra is indicated for the treatment of active systemic juvenile idiopathic arthritis (sJIA) in patients 2 years of age and older, who have responded inadequately to previous therapy with NSAIDs and systemic corticosteroids. RoActemra can be given as monotherapy (in case of intolerance to MTX or where treatment with MTX is inappropriate) or in combination with MTX.

RoActemra in combination with methotrexate (MTX) is indicated for the treatment of juvenile idiopathic polyarthritis (pJIA; rheumatoid factor positive or negative and extended oligoarthritis) in patients 2 years of age and older, who have responded inadequately to previous therapy with MTX. RoActemra can be given as monotherapy in case of intolerance to MTX or where continued treatment with MTX is inappropriate.

RoActemra is indicated for the treatment of chimeric antigen receptor (CAR) T cell-induced severe or life-threatening cytokine release syndrome (CRS) in adults and paediatric patients 2 years of age and older.

4.2 Posology and method of administration

Treatment should be initiated by healthcare professionals experienced in the diagnosis and treatment of RA, sJIA, pJIA or CRS.

All patients treated with RoActemra should be given the Patient Alert Card.

<u>Posology</u>
<u>RA Patients</u>
The recommended posology is 8 mg/kg body weight, given once every four weeks.

For individuals whose body weight is more than 100 kg, doses exceeding 800 mg per infusion are not recommended (see section 5.2).

Doses above 1.2 g have not been evaluated in clinical

studies (see section 5.1). <u>Dose adjustments due to</u>

<u>laboratory abnormalities (see section 4.4).</u>

- Liver enzyme abnormalities

Laboratory Value	Action
> 1 to 3 x Upper Limit of Normal (ULN)	Modify the dose of the concomitant MTX if appropriate<br>For persistent increases in this range, reduce RoActemra dose to 4 mg/kg or interrupt RoActemra until alanine aminotransferase (ALT) or aspartate aminotransferase (AST) have normalised<br>Restart with 4 mg/kg or 8 mg/kg, as clinically appropriate
> 3 to 5 x ULN<br>(confirmed by repeat testing, see section 4.4).	Interrupt RoActemra dosing until < 3 x ULN and follow recommendations above for > 1 to 3 x ULN<br>For persistent increases > 3 x ULN, discontinue RoActemra
> 5 x ULN	Discontinue RoActemra

- Low absolute neutrophil count (ANC)

In patients not previously treated with RoActemra, initiation is not recommended in patients with an absolute neutrophil count (ANC) below 2 x 10^9/l.

Laboratory Value (cells x 10^9/ l)	Action
ANC > 1	Maintain dose
ANC 0.5 to 1	Interrupt RoActemra dosing<br>When ANC increases > 1 x 10^9/ l resume RoActemra at 4 mg/kg and increase to 8 mg/kg as clinically appropriate
ANC < 0.5	Discontinue RoActemra

- Low platelet count

Laboratory Value (cells x 10^3/ µl)	Action

50 to 100	Interrupt RoActemra dosing<br>When platelet count > 100 x 10^3/ µ resume RoActemra at 4 mg/kg and increase to 8 mg/kg as clinically appropriate
< 50	Discontinue RoActemra

Cytokine Release Syndrome (CRS) (adults and paediatrics)

The recommended posology for treatment of CRS given as a 60-minute intravenous infusion is

8 mg/kg in patients weighing greater than or equal to 30 kg or 12 mg/kg in patients weighing less than 30 kg. RoActemra can be given alone or in combination with corticosteroids.

If no clinical improvement in the signs and symptoms of CRS occurs after the first dose, up to 3 additional doses of RoActemra may be administered. The interval between consecutive doses should be at least 8 hours. Doses exceeding 800 mg per infusion are not recommended in CRS patients.

Patients with severe or life-threatening CRS frequently have cytopenias or elevated ALT or AST due to the underlying malignancy, preceding lymphodepleting chemotherapy or the CRS.

Special populations

Paediatric patients

sJIA Patients

The recommended posology in patients above 2 years of age is 8 mg/kg once every 2 weeks in patients weighing greater than or equal to 30 kg or 12 mg/kg once every 2 weeks in patients weighing less than 30 kg. The dose should be calculated based on the patient's body weight at each administration. A change in dose should only be based on a consistent change in the patient's body weight over time.

The safety and efficacy of intravenous RoActemra in children below 2 years of age has not been established.

Dose interruptions of tocilizumab for the following laboratory abnormalities are recommended in sJIA patients in the tables below. If appropriate, the dose of concomitant MTX and/or other medications should be modified or dosing stopped and tocilizumab dosing interrupted until the clinical situation has been evaluated. As there are many co-morbid conditions that may affect laboratory values in sJIA, the decision to discontinue tocilizumab for a laboratory abnormality should be based upon the medical assessment of the individual patient.

- Liver enzyme abnormalities

**Laborator y Value**	**Actio n**
> 1 to 3 x ULN	Modify the dose of the concomitant MTX if appropriate<br>For persistent increases in this range, interrupt RoActemra until ALT/AST have normalized.
> 3 x ULN to 5x ULN	Modify the dose of the concomitant MTX if appropriate<br>Interrupt RoActemra dosing until < 3x ULN and follow recommendations above for >1 to 3x ULN
> 5x ULN	Discontinue RoActemra.<br>The decision to discontinue RoActemra in sJIA for a laboratory abnormality should be based on the medical assessment of the individual patient.

- Low absolute neutrophil count (ANC)

**Laborator y Value (cells x 10^9/ l)**	**Actio n**
ANC > 1	Maintain dose
ANC 0.5 to 1	Interrupt RoActemra dosing<br>When ANC increases to > 1 x 10^9/ l resume RoActemra
ANC < 0.5	Discontinue RoActemra<br>The decision to discontinue RoActemra in sJIA for a laboratory abnormality should be based on the medical assessment of the individual patient.

- Low platelet count

**Laboratory Value (cells x [illegible]**	**Actio n**

50 to 100	Modify the dose of the concomitant MTX if<br>appropriate Interrupt RoActemra dosing<br>When platelet count is > 100 x $10^3/\mu l$ resume RoActemra
< 50	Discontinue RoActemra.<br>The decision to discontinue RoActemra in sJIA for a laboratory abnormality should be based on the medical assessment of the individual patient.

Reduction of tocilizumab dose due to laboratory abnormalities has not been studied in sJIA patients.

Available data suggest that clinical improvement is observed within 6 weeks of initiation of treatment with RoActemra. Continued therapy should be carefully reconsidered in a patient exhibiting no improvement within this timeframe.

pJIA Patients

The recommended posology in patients above 2 years of age is 8 mg/kg once every 4 weeks in patients weighing greater than or equal to 30 kg or 10 mg/kg once every 4 weeks in patients weighing less than 30 kg. The dose should be calculated based on the patient's body weight at each administration. A change in dose should only be based on a consistent change in the patient's body weight over time.

The safety and efficacy of intravenous RoActemra in children below 2 years of age has not been established.

Dose interruptions of tocilizumab for the following laboratory abnormalities are recommended in pJIA patients in the tables below. If appropriate, the dose of concomitant MTX and/or other medications should be modified or dosing stopped and tocilizumab dosing interrupted until the clinical situation has been evaluated. As there are many co-morbid conditions that may effect laboratory values in pJIA, the decision to discontinue tocilizumab for a laboratory abnormality should be based upon the medical assessment of the individual patient.

- Liver enzyme abnormalities

**Laborator y Value**	**Actio n**

> 1 to 3 x ULN	Modify the dose of the concomitant MTX if appropriate<br>For persistent increases in this range, interrupt RoActemra until ALT/AST have normalized.
> 3 x ULN to 5x ULN	Modify the dose of the concomitant MTX if appropriate<br>Interrupt RoActemra dosing until < 3x ULN and follow recommendations above for >1 to 3x ULN
> 5x ULN	Discontinue RoActemra.<br>The decision to discontinue RoActemra in pJIA for a laboratory abnormality should be based on the medical assessment of the individual patient.

- Low absolute neutrophil count (ANC)

**Laboratory Value (cells x 10^9/ l)**	**Action**
ANC > 1	Maintain dose
ANC 0.5 to 1	Interrupt RoActemra dosing<br>When ANC increases to > 1 x 10^9/ l resume RoActemra
ANC < 0.5	Discontinue RoActemra<br>The decision to discontinue RoActemra in pJIA for a laboratory abnormality should be based on the medical assessment of the individual patient.

- Low platelet count

**Laboratory Value (cells x 10^3/µl)**	**Action**
50 to 100	Modify the dose of the concomitant MTX if<br>appropriate Interrupt RoActemra dosing<br>When platelet count is > 100 x 10^3/µl resume RoActemra

< 50	Discontinue RoActemra.<br><br>The decision to discontinue RoActemra in pJIA for a laboratory abnormality should be based on the medical assessment of the individual patient.

Reduction of tocilizumab dose due to laboratory abnormalities has not been studied in pJIA patients.

Available data suggest that clinical improvement is observed within 12 weeks of initiation of treatment with RoActemra. Continued therapy should be carefully reconsidered in a patient exhibiting no improvement within this timeframe.

Elderly

No dose adjustment is required in elderly patients >65 years of age.

Renal impairment

No dose adjustment is required in patients with mild renal impairment. RoActemra has not been studied in patients with moderate to severe renal impairment (see section 5.2). Renal function should be monitored closely in these patients.

Hepatic impairment

RoActemra has not been studied in patients with hepatic impairment. Therefore, no dose recommendations can be made.

<u>Method of administration</u>

After dilution, RoActemra for RA, sJIA, pJIA, and CRS patients should be administered as an intravenous infusion over 1 hour.

RA, sJIA, pJIA and CRS Patients ≥ 30 kg

RoActemra should be diluted to a final volume of 100 mL with sterile, non-pyrogenic sodium chloride 9 mg/mL (0.9%) solution for injection using aseptic technique.

For instructions on dilution of the medicinal product before

administration, see section 6.6. sJIA,pJIA and CRS Patients < 30 kg

RoActemra should be diluted to a final volume of 50 mL with sterile, non-pyrogenic sodium chloride

9 mg/mL (0.9%) solution for injection using aseptic technique.

For instructions on dilution of the medicinal product before administration, see section 6.6.

4.3 Contraindications

Hypersensitivity to the active substance or to any of the excipients

listed in section 6.1. Active, severe infections (see section 4.4).

4.4 Special warnings and precautions for use

Traceability

In order to improve the traceability of biological medicinal products, the name and the batch number of the administered product should be clearly recorded.

Infections

Serious and sometimes fatal infections have been reported in patients receiving immunosuppressive agents including RoActemra (see section 4.8, undesirable effects). RoActemra treatment must not be initiated in patients with active infections (see section 4.3). Administration of RoActemra should be interrupted if a patient develops a serious infection until the infection is controlled (see section 4.8). Healthcare professionals should exercise caution when considering the use of RoActemra in patients with a history of recurring or chronic infections or with underlying conditions (e.g. diverticulitis, diabetes and interstitial lung disease which may predispose patients to infections.

Vigilance for the timely detection of serious infection is recommended for patients receiving biological treatments for moderate to severe RA, sJIA or pJIA as signs and symptoms of acute inflammation may be lessened, associated with suppression of the acute phase reaction. The effects of tocilizumab on C-reactive protein (CRP), neutrophils and signs and symptoms of infection should be considered when evaluating a patient for a potential infection. Patients (which includes younger children with sJIA or pJIA who may be less able to communicate their symptoms) and parents/guardians of sJIA or pJIA patients, should be instructed to contact their healthcare professional immediately when any symptoms suggesting infection appear, in order to assure rapid evaluation and appropriate treatment.

Tuberculosis

As recommended for other biological treatments, RA, sJIA and pJIA patients should be screened for latent tuberculosis (TB) infection prior to starting RoActemra therapy. Patients with latent TB should be treated with standard anti-mycobacterial therapy before initiating RoActemra. Prescribers are reminded of the risk of false negative tuberculin skin and interferon-gamma TB blood test results, especially in patients who are severely ill or immunocompromised.

Patients should be instructed to seek medical advice if signs/symptoms (e.g., persistent cough, wasting/weight loss, low grade fever) suggestive of a tuberculosis infection occur during or after therapy with RoActemra.

Viral reactivation

Viral reactivation (e.g. hepatitis B virus) has been reported with biologic therapies for RA. In clinical studies with tocilizumab, patients who screened positive for hepatitis were excluded.

Complications of diverticulitis

Events of diverticular perforations as complications of diverticulitis have been reported uncommonly with RoActemra in RA patients (see section 4.8). RoActemra should be used with caution in patients with previous history of intestinal ulceration or diverticulitis. Patients presenting with symptoms potentially indicative of complicated diverticulitis, such as abdominal pain, haemorrhage and/or unexplained change in bowel habits with fever should be evaluated promptly for early identification of diverticulitis which can be associated with gastrointestinal perforation.

Hypersensitivity reactions

Serious hypersensitivity reactions have been reported in association with infusion of RoActemra (see section 4.8). Such reactions may be more severe, and potentially fatal in patients who have experienced hypersensitivity reactions during previous infusions even if they have received premedication with steroids and antihistamines. Appropriate treatment should be available for immediate use in the event of an anaphylactic reaction during treatment with RoActemra. If an anaphylactic reaction or other serious hypersensitivity / serious infusion related reaction occurs, administration of RoActemra should be stopped immediately and RoActemra should be permanently discontinued.

Active hepatic disease and hepatic impairment

Treatment with RoActemra, particularly when administered concomitantly with MTX, may be associated with elevations in hepatic transaminases, therefore, caution should be exercised when considering treatment of patients with active hepatic disease or hepatic impairment (see sections 4.2 and 4.8).

Hepatotoxicity

Transient or intermittent mild and moderate elevations of hepatic transaminases have been reported commonly with RoActemra treatment (see section 4.8). An increased frequency of these elevations was observed when potentially hepatotoxic drugs (e.g. MTX) were used in combination with RoActemra. When clinically indicated, other liver function tests including bilirubin should be considered.

Serious drug-induced liver injury, including acute liver failure, hepatitis and jaundice, have been observed with RoActemra (see section 4.8). Serious hepatic injury occurred between 2 weeks to more than 5 years after initiation of RoActemra. Cases of liver failure resulting in liver transplantation have been

reported. Patients should be advised to immediately seek medical help if they experience signs and symptoms of hepatic injury.

Caution should be exercised when considering initiation of RoActemra treatment in patients with elevated ALT or AST > 1.5 x ULN. In patients with baseline ALT or AST > 5 x ULN, treatment is not recommended.

In RA, pJIA and sJIA patients, ALT/AST should be monitored every 4 to 8 weeks for the first 6 months of treatment followed by every 12 weeks thereafter. For recommended modifications, including RoActemra discontinuation, based on transaminases levels see section 4.2. For ALT or AST elevations > 3–5 x ULN, confirmed by repeat testing, RoActemra treatment should be interrupted.

Haematological abnormalities

Decreases in neutrophil and platelet counts have occurred following treatment with tocilizumab

8 mg/kg in combination with MTX (see section 4.8). There may be an increased risk of neutropenia in patients who have previously been treated with a TNF antagonist.

In patients not previously treated with RoActemra, initiation is not recommended in patients with an absolute neutrophil count (ANC) below 2 x 10^9/l. Caution should be exercised when considering initiation of RoActemra treatment in patients with a low platelet count (i.e. platelet count below

100 x 10^3/ μL). In patients who develop an ANC < 0.5 x 10^9/ l or a platelet count < 50 x 10^3/μL, continued treatment is not recommended.

Severe neutropenia may be associated with an increased risk of serious infections, although there has been no clear association between decreases in neutrophils and the occurrence of serious infections in clinical trials with RoActemra to date.

In RA patients, neutrophils and platelets should be monitored 4 to 8 weeks after start of therapy and thereafter according to standard clinical practice. For recommended dose modifications based on ANC and platelet counts, see section 4.2.

In sJIA and pJIA patients, neutrophils and platelets should be monitored at the time of second infusion and thereafter according to good clinical practice, see section 4.2.

Lipid parameters

Elevations in lipid parameters including total cholesterol, low-density lipoprotein (LDL), high-density lipoprotein (HDL) and triglycerides were observed in patients treated with tocilizumab (see

section 4.8). In the majority of patients, there was no increase in atherogenic indices, and elevations in total cholesterol responded to treatment with lipid lowering agents.

In sJIA, pJIA and RA patients, assessment of lipid parameters should be performed 4 to 8 weeks following initiation of RoActemra therapy. Patients should be managed according to local clinical guidelines for management of hyperlipidaemia.

Neurological disorders

Physicians should be vigilant for symptoms potentially indicative of new-onset central demyelinating disorders. The potential for central demyelination with RoActemra is currently unknown.

Malignancy

The risk of malignancy is increased in patients with RA. Immunomodulatory medicinal products may increase the risk of malignancy.

Vaccinations

Live and live attenuated vaccines should not be given concurrently with RoActemra as clinical safety has not been established. In a randomized open-label study, adult RA patients treated with RoActemra and MTX were able to mount an effective response to both the 23-valent pneumococcal polysaccharide and tetanus toxoid vaccines which was comparable to the response seen in patients on MTX only. It is recommended that all patients, particularly sJIA and pJIA patients, be brought up to date with all immunisations in agreement with current immunisation guidelines prior to initiating RoActemra therapy. The interval between live vaccinations and initiation of RoActemra therapy should be in accordance with current vaccination guidelines regarding immunosuppressive agents.

Cardiovascular risk

RA patients have an increased risk for cardiovascular disorders and should have risk factors (e.g. hypertension, hyperlipidaemia) managed as part of usual standard of care.

Combination with TNF antagonists

There is no experience with the use of RoActemra with TNF antagonists or other biological treatments for RA, sJIA or pJIA patients. RoActemra is not recommended for use with other biological agents.

Sodium

This medicinal product contains 1.17 mmol (or 26.55 mg) sodium per maximum dose of 1200 mg. To be taken into consideration by patients on a controlled sodium diet. Doses below 1025 mg of this medicinal product contain less than 1 mmol sodium (23 mg), i.e. essentially ‘sodium free’.

<u>Paediatric population</u>

sJIA Patients

Macrophage activation syndrome (MAS) is a serious life-threatening disorder that may develop in sJIA patients. In clinical trials, tocilizumab has not been studied in patients during an episode of active MAS.

4.5 Interaction with other medicinal products and other forms of interaction

Interaction studies have only been performed in adults.

Concomitant administration of a single dose of 10 mg/kg tocilizumab with 10-25 mg MTX once weekly had no clinically significant effect on MTX exposure.

Population pharmacokinetic analyses did not detect any effect of MTX, non-steroidal anti- inflammatory drugs (NSAIDs) or corticosteroids on tocilizumab clearance.

The expression of hepatic CYP450 enzymes is suppressed by cytokines, such as IL-6, that stimulate chronic inflammation. Thus, CYP450 expression may be reversed when potent cytokine inhibitory therapy, such as tocilizumab, is introduced.

In vitro **studies with cultured human hepatocytes demonstrated that IL-6 caused a reduction in CYP1A2, CYP2C9, CYP2C19 and CYP3A4 enzyme expression. Tocilizumab normalises expression of these enzymes.**

In a study in RA patients, levels of simvastatin (CYP3A4) were decreased by 57% one week following a single dose of tocilizumab, to the level similar to, or slightly higher than, those observed in healthy subjects.

When starting or stopping therapy with tocilizumab, patients taking medicinal products which are individually adjusted and are metabolised via CYP450 3A4, 1A2 or 2C9 (e.g. . methylprednisolone, dexamethasone, (with the possibility for oral glucocorticoid withdrawal syndrome), atorvastatin, calcium channel blockers, theophylline, warfarin, phenprocoumon, phenytoin, ciclosporin, or benzodiazepines) should be monitored as doses may need to be increased to maintain therapeutic effect. Given its long elimination half-life ($t_{1/2}$), the effect of tocilizumab on CYP450 enzyme activity may persist for several weeks after stopping therapy.

4.6 Fertility, pregnancy and lactation

<u>Women of childbearing potential</u>

Women of childbearing potential must use effective contraception during and up to 3 months after treatment.

Pregnancy

There are no adequate data from the use of tocilizumab in pregnant women. A study in animals has shown an increased risk of spontaneous abortion/embryo-foetal death at a high dose (see section 5.3). The potential risk for humans is unknown.

RoActemra should not be used during pregnancy

unless clearly necessary. Breast-feeding

It is unknown whether tocilizumab is excreted in human breast milk. The excretion of tocilizumab in milk has not been studied in animals. A decision on whether to continue/discontinue breast-feeding or to continue/discontinue therapy with RoActemra should be made taking into account the benefit of breast-feeding to the child and the benefit of RoActemra therapy to the woman.

Fertility

Available non-clinical data do not suggest an effect on fertility under tocilizumab treatment.

4.7 Effects on ability to drive and use machines

RoActemra has minor influence on the ability to drive and use machines (see section 4.8, dizziness).

4.8 Undesirable effects

Summary of the safety profile

The most commonly reported ADRs (occurring in ≥ 5% of patients treated with tocilizumab monotherapy or in combination with DMARDs) were upper respiratory tract infections, nasopharyngitis, headache, hypertension and increased ALT.

The most serious ADRs were serious infections, complications of diverticulitis, and hypersensitivity reactions.

RA Patients

The safety profile of tocilizumab has been studied in 4 placebo-controlled studies (studies II, III, IV and V), 1 MTX-controlled study (study I) and their extension periods (see section 5.1).

The double-blind controlled period was 6 months in four studies (studies I, III, IV and V) and was up to 2 years in one study (study II). In the double-blind controlled studies, 774 patients received tocilizumab 4 mg/kg in combination with MTX, 1870 patients received tocilizumab 8 mg/kg in combination with MTX or other DMARDs and 288 patients received tocilizumab 8 mg/kg monotherapy.

The long-term exposure population includes all patients who received at least one dose of tocilizumab either in the double-blind control period or open label extension phase in the studies. Of the 4009 patients in this population, 3577 received treatment for at least 6 months, 3296 for at least one year, 2806 received treatment for at least 2 years and 1222 for 3 years.

ADRs from clinical trials and/or post marketing experience with RoActemra based on spontaneous case reports, literature cases and cases from non-interventional study programs are listed in Table 1 and are presented by MedDRA system organ class. The corresponding frequency category for each ADR is based on the following convention: very common (≥ 1/10); common (≥ 1/100 to < 1/10), uncommon (≥ 1/1,000 to < 1/100), rare (>1/10,000 to <1/1,000) or very rare (<1/10,000). Within each frequency grouping, undesirable effects are presented in order of decreasing seriousness.

Table 1. List of ADRs occurring in patients with RA receiving tocilizumab as monotherapy or in combination with MTX or other DMARDs in the double-blind controlled period or during postmarketing experience

MedDRA System Organ Class	Frequency categories with preferred terms			
	**Very Common**	**Common**	**Uncommon**	**Rare**
Infections and infestations	Upper respiratory tract infections	Cellulitis, Pneumonia, Oral herpes simplex,	Diverticulitis	
Gastrointestin al disorders		Abdominal pain, Mouth ulceration,	Stomatitis, Gastric ulcer	
Skin and subcutaneous tissue		Rash, Pruritus, Urticaria		Stevens-Johnson-Syndrome[3]
Nervous system disorders		Headach e,		
Investigations		Hepatic transaminases increased, Weight increased, Total bilirubin		
Vascula r		Hypertension		
Blood and lymphatic system disorders		Leukopenia, Neutropenia, Hypofibrinogenae m ia		
Immune system				Anaphylaxi s (fatal)[1, 2]
Metabolism and nutrition disorders	Hypercholesterol ae mia*		Hypertriglycerida e mia	
General disorders and administratio		Peripheral oedema, Hypersensitivity reactions		
Eye disorders		Conjunctivitis		

Respiratory, thoracic and mediastina		Cough, Dyspnoea		
Renal disorders			Nephrolithiasis	
Endocrine			Hypothyroidism	
Hepatobiliary disorders				Drug-induced liver injury, Hepatitis, Jaundice, Very rare:

* Includes elevations collected as part of routine laboratory monitoring (see text below)

[1] See section 4.3

[2] See section 4.4

[3] This adverse reaction was identified through post marketing surveillance but not observed in controlled clinical trials. The frequency category was estimated as the upper limit of the 95% confidence interval calculated on the basis of the total number of patients exposed to TCZ in clinical trials.

Infections

In the 6-month controlled studies the rate of all infections reported with tocilizumab 8 mg/kg plus DMARD treatment was 127 events per 100 patient years compared to 112 events per 100 patient years in the placebo plus DMARD group. In the long-term exposure population, the overall rate of infections with RoActemra was 108 events per 100 patient years exposure.

In 6-month controlled clinical studies, the rate of serious infections with tocilizumab 8 mg/kg plus DMARDs was 5.3 events per 100 patient years exposure compared to 3.9 events per 100 patient years exposure in the placebo plus DMARD group. In the monotherapy study the rate of serious infections was 3.6 events per 100 patient years of exposure in the tocilizumab group and 1.5 events per 100 patient years of exposure in the MTX group.

In the long-term exposure population, the overall rate of serious infections (bacterial, viral and fungal) was 4.7 events per 100 patient years. Reported serious infections, some with fatal outcome, included active tuberculosis, which may present with intrapulmonary or extrapulmonary disease, invasive pulmonary infections, including candidiasis, aspergillosis, coccidioidomycosis and pneumocystis jirovecii, pneumonia, cellulitis, herpes zoster, gastroenteritis, diverticulitis, sepsis and bacterial arthritis. Cases of opportunistic infections have been reported.

Interstitial Lung Disease

Impaired lung function may increase the risk for developing infections. There have been post- marketing reports of interstitial lung disease (including pneumonitis and pulmonary fibrosis), some of which had fatal outcomes.

Gastrointestinal Perforation

During the 6-month controlled clinical trials, the overall rate of gastrointestinal perforation was 0.26 events per 100 patient years with tocilizumab therapy. In the long-term exposure population the overall rate of gastrointestinal perforation was 0.28 events per 100 patient years. Reports of gastrointestinal perforation on tocilizumab were primarily reported as complications of diverticulitis including generalised purulent peritonitis, lower gastrointestinal perforation, fistulae and abscess.

Infusion reactions

In the 6-month controlled trials adverse events associated with infusion (selected events occurring during or within 24 hours of infusion) were reported by 6.9% of patients in the tocilizumab 8 mg/kg plus DMARD group and 5.1% of patients in the placebo plus DMARD group. Events reported during the infusion were primarily episodes of hypertension; events reported within 24 hours of finishing an infusion were headache and skin reactions (rash, urticaria). These events were not treatment limiting.

The rate of anaphylactic reactions (occurring in a total of 8/4,009 patients, 0.2%) was several fold higher with the 4 mg/kg dose, compared to the 8 mg/kg dose. Clinically significant hypersensitivity reactions associated with tocilizumab and requiring treatment discontinuation were reported in a total of 56 out of 4,009 patients (1.4%) treated with tocilizumab during the controlled and open label clinical studies. These reactions were generally observed during the second to fifth infusions of tocilizumab (see section 4.4). Fatal anaphylaxis has been reported after marketing authorisation during treatment with tocilizumab (see section 4.4).

Immunogenicity

A total of 2,876 patients have been tested for anti-tocilizumab antibodies in the 6-month controlled clinical trials. Of the 46 patients (1.6%) who developed anti-tocilizumab antibodies, 6 had an associated medically significant hypersensitivity reaction, of which 5 led to permanent discontinuation of treatment. Thirty patients (1.1%) developed neutralising antibodies.

Haematological abnormalities:
Neutrophils

In the 6-month controlled trials decreases in neutrophil counts below 1×10^9/ l occurred in 3.4% of patients on tocilizumab 8 mg/kg plus DMARDs compared to < 0.1% of patients on placebo plus DMARDs. Approximately half of the patients who developed an ANC < 1×10^9/ l did so within 8 weeks after starting therapy. Decreases below 0.5×10^9/ l were reported in 0.3% patients receiving tocilizumab 8 mg/kg plus DMARDs. Infections with neutropenia have been reported.

During the double-blind controlled period and with long-term exposure, the pattern and incidence of decreases in neutrophil counts remained consistent with what was seen in the 6-month controlled clinical trials.

Platelets

In the 6-month controlled trials decreases in platelet counts below 100 x 10^3/ µL occurred in 1.7% of patients on tocilizumab 8 mg/kg plus DMARDs compared to < 1% on placebo plus DMARDs. These decreases occurred without associated bleeding events.

During the double-blind controlled period and with long-term exposure, the pattern and incidence of decreases in platelet counts remained consistent with what was seen in the 6-month controlled clinical trials.

Very rare reports of pancytopenia have occurred in the post marketing setting.

Hepatic transaminase elevations

During the 6-month controlled trials transient elevations in ALT/AST > 3 x ULN were observed in 2.1% of patients on tocilizumab 8 mg/kg compared to 4.9% of patients on MTX and in 6.5% of patients who received 8 mg/kg tocilizumab plus DMARDs compared to 1.5% of patients on placebo plus DMARDs.

The addition of potentially hepatotoxic drugs (e.g. MTX) to tocilizumab monotherapy resulted in increased frequency of these elevations. Elevations of ALT/AST > 5 x ULN were observed in 0.7% of tocilizumab monotherapy patients and 1.4% of tocilizumab plus DMARD patients, the majority of whom were discontinued permanently from tocilizumab treatment. During the double-blind controlled period, the incidence of indirect bilirubin greater than the upper limit of normal, collected as a routine laboratory parameter, is 6.2% in patients treated with 8 mg/kg tocilizumab + DMARD. A total of 5.8% of patients experienced an elevation of indirect bilirubin of > 1 to 2 x ULN and 0.4% had an elevation of > 2 x ULN.

During the double-blind controlled period and with long-term exposure, the pattern and incidence of elevation in ALT/AST remained consistent with what was seen in the 6-month controlled clinical trials.

Lipid parameters

During the 6-month controlled trials, increases of lipid parameters such as total cholesterol, triglycerides, LDL cholesterol, and/or HDL cholesterol have been reported commonly. With routine laboratory monitoring it was seen that approximately 24% of patients receiving RoActemra in clinical trials experienced sustained elevations in total cholesterol ≥ 6.2 mmol/ l, with 15% experiencing a sustained increase in LDL to ≥ 4.1 mmol/ l. Elevations in lipid parameters responded to treatment with lipid-lowering agents.

During the double-blind controlled period and with long-term exposure, the pattern and incidence of elevations in lipid parameters remained consistent with what was seen in the 6-month controlled trials.

Malignancies

The clinical data are insufficient to assess the potential incidence of malignancy following exposure to tocilizumab. Long-term safety evaluations are ongoing.

Skin Reactions

Rare reports of Stevens-Johnson Syndrome have occurred in the post marketing setting.

sJIA and pJIA Patients

The safety profile of tocilizumab in the pediatric population is summarized in the sections on pJIA and sJIA below. In general, the ADRs in pJIA and sJIA patients were similar in type to those seen in RA patients, see section 4.8.

ADRs in the pJIA and sJIA patients treated with tocilizumab are listed in the Table 2 and presented by MedDRA system organ class. The corresponding frequency category for each ADR is based on the following convention: very common (≥ 1/10); common (≥ 1/100 to < 1/10) or uncommon (≥ 1/1,000 to <1/100).

Table 2: List of ADRs occurring in clinical trial patients with sJIA or pJIA receiving tocilizumab as monotherapy or in combination with MTX.

**MedDRA SOC**	**Preferrred term (PT)**	**Frequenc**		
Infections and Infestations		Very Common	Common	Uncommon
	Upper Respiratory	pJIA, sJIA		
	Nasopharyngitis	pJIA, sJIA		
Gastrointestinal Disorders				
	Nausea		pJIA	
	Diarrhea		pJIA, sJIA	
General disorders and administration site conditions				
	Infusion related		pJIA[1], sJIA[2]	
Nervous system disorders				
	Headache	pJIA	sJIA	
Investigations				
	Hepatic transaminases increased		pJIA	
	Decrease in neutrophil count	sJIA	pJIA	
	Platelet count		sJIA	pJIA
	Cholesterol increased		sJIA	pJIA

1. Infusion related reaction events in pJIA patients included but were not limited to headache, nausea and hypotension
2. Infusion related reaction events in sJIA patients included but were not limited to rash, urticaria, diarrhoea, epigastric discomfort, arthralgia and headache

pJIA Patients

The safety profile of intravenous RoActemra in pJIA has been studied in 188 patients from 2 to 17 years of age. The total patient exposure was 184.4 patient years. The frequency of ADRs in pJIA patients can be found in Table 2. The types of ADRs in pJIA patients were similar to those seen in RA and sJIA patients, see section 4.8. When compared to the adult RA population, events of

nasopharyngitis, headache, nausea, and decreased neutrophil count were more frequently reported in the pJIA population. Events of cholesterol increased were less frequently reported in the pJIA population than in the adult RA population.

Infections

The rate of infections in the tocilizumab all exposure population was 163.7 per 100 patient years. The most common events observed were nasopharyngitis and upper respiratory tract infections. The rate of serious infections was numerically higher in patients weighing <30 kg treated with 10 mg/kg tocilizumab (12.2 per 100 patient years) compared to patients weighing ≥30 kg, treated with 8 mg/kg tocilizumab (4.0 per 100 patient years). The incidence of infections leading to dose interruptions was also numerically higher in patients weighing <30 kg treated with 10 mg/kg tocilizumab (21.4%) compared to patients weighing ≥30 kg, treated with 8 mg/kg tocilizumab (7.6%).

Infusion Reactions

In pJIA patients, infusion related reactions are defined as all events occurring during or within 24 hours of an infusion. In the tocilizumab all exposure population, 11 patients (5.9%) experienced infusion reactions during the infusion and 38 patients (20.2%) experienced an event within 24 hours of an infusion. The most common events occurring during infusion were headache, nausea and hypotension and within 24 hours of infusion were dizziness and hypotension. In general, the adverse drug reactions observed during or within 24 hours of an infusion were similar in nature to those seen in RA and sJIA patients, see section 4.8.

No clinically significant hypersensitivity reactions associated with tocilizumab and requiring treatment discontinuation were reported.

Immunogenicity

One patient in the 10 mg/kg < 30kg group developed positive anti-tocilizumab antibodies without developing a hypersensitivity reaction and subsequently withdrew from the study.

Neutrophils

During routine laboratory monitoring in the tocilizumab all exposure population, a decrease in neutrophil count below 1 × 10^9/L occurred in 3.7% of patients.

Platelets

During routine laboratory monitoring in the tocilizumab all exposure population, 1% of patients had a decrease in platelet count to ≤ 50 × 10^3/μL without associated bleeding events.

Hepatic transaminase elevations

During routine laboratory monitoring in the tocilizumab all exposure population, elevation in ALT or AST ≥ 3xULN occurred in 3.7% and <1% of patients, respectively.

Lipid parameters

During routine laboratory monitoring in the intravenous RoActemra study WA19977 3.4% and 10.4% of patients experienced a post-baseline elevation of their LDL-cholesterol value to ≥ 130 mg/dL and total cholesterol value to ≥ 200 mg/dL at any time during the study treatment, respectively.

sJIA Patients

The safety profile of intravenous RoActemra in sJIA has been studied in 112 patients from 2 to 17 years of age. In the 12 week double-blind, controlled phase, 75 patients received treatment with tocilizumab (8 mg/kg or 12 mg/kg based upon body weight). After 12 weeks or at the time of switching to RoActemra, due to disease worsening, patients were treated in the open label extension phase.

In general, the ADRs in sJIA patients were similar in type to those seen in RA patients, see section

4.8. The frequency of ADRs in sJIA patients can be found in Table 2. When compared to the adult RA population, patients with sJIA experienced a higher frequency of nasopharyngitis, decrease in neutrophil counts, hepatic transaminases increased, and diarrhea. Events of cholesterol increased were less frequently reported in the sJIA population than in the adult RA population.

Infections

In the 12 week controlled phase, the rate of all infections in the intravenous RoActemra group was

344.7 per 100 patient years and 287.0 per 100 patient years in the placebo group. In the open label extension phase (Part II), the overall rate of infections remained similar at 306.6 per 100 patient years.

In the 12 week controlled phase, the rate of serious infections in the intravenous RoActemra group was 11.5 per 100 patient years. At one year in the open label extension phase the overall rate of serious infections remained stable at 11.3 per 100 patient years. Reported serious infections were similar to those seen in RA patients with the addition of varicella and otitis media.

Infusion Reactions

Infusion related reactions are defined as all events occurring during or within 24 hours of an infusion. In the 12 week controlled phase, 4% of patients from the tocilizumab group experienced events occurring during infusion. One event (angioedema) was considered serious and life-threatening, and the patient was discontinued from study treatment.

In the 12 week controlled phase, 16% of patients in the tocilizumab group and 5.4% of patients in the placebo group experienced an event within 24 hours of infusion. In the tocilizumab group, the events included, but were not limited to rash, urticaria, diarrhea, epigastric discomfort, arthralgia and headache. One of these events, urticaria, was considered serious.

Clinically significant hypersensitivity reactions associated with tocilizumab and requiring treatment discontinuation, were reported in 1 out of 112 patients (< 1%) treated with tocilizumab during the controlled and up to and including the open label clinical trial.

Immunogenicity

All 112 patients were tested for anti-tocilizumab antibodies at baseline. Two patients developed positive anti-tocilizumab antibodies with one of these patients having a hypersensitivity reaction leading to withdrawal. The incidence of anti-tocilizumab antibody formation might be underestimated because of interference of tocilizumab with the assay and higher drug concentration observed in children compared to adults.

Neutrophils

During routine laboratory monitoring in the 12 week controlled phase, a decrease in neutrophil counts below 1 x 10^9/l occurred in 7% of patients in the tocilizumab group, and no decreases in the placebo group.

In the open label extension phase, decreases in neutrophil counts below 1 x 10^9/l, occurred in 15% of the tocilizumab group.

Platelets

During routine laboratory monitoring in the 12 week controlled phase, 3% of patients in the placebo group and 1% in the tocilizumab group had a decrease in platelet count to ≤ 100 x 10^3/μl.

In the open label extension phase, decreases in platelet counts below 100 x 10^3/μl, occurred in 3% of patients in the tocilizumab group, without associated bleeding events.

Hepatic transaminase elevations

During routine laboratory monitoring in the 12 week controlled phase, elevation in ALT or AST ≥ 3 x ULN occurred in 5% and 3% of patients, respectively, in the tocilizumab group, and 0% in the placebo group.

In the open label extension phase, elevation in ALT or AST ≥ 3 x ULN occurred in 12% and 4% of patients, respectively, in the tocilizumab group.

Immunoglobulin G

IgG levels decrease during therapy. A decrease to the lower limit of normal occurred in 15 patients at some point in the study.

Lipid parameters

During routine laboratory monitoring in the 12 week controlled phase (study WA18221), 13.4% and 33.3% of patients experienced a post-baseline elevation of their LDL-cholesterol value to ≥ 130 mg/dL and total cholesterol value to ≥ 200 mg/dL at any time during study treatment, respectively.

In the open label extension phase (study WA18221), 13.2% and 27.7% of patients experienced a post- baseline elevation of their LDL-cholesterol value to ≥ 130 mg/dL and total cholesterol value to ≥ 200 mg/dL at any time during study treatment, respectively.

CRS Patients

The safety of tocilizumab in CRS has been evaluated in a retrospective analysis of data from clinical trials, where 51 patients were treated with intravenous tocilizumab 8 mg/kg (12 mg/kg for patients less than 30 kg) with or without additional high-dose corticosteroids for severe or life-threatening CAR T- cell-induced CRS. A median of 1 dose of tocilizumab (range, 1-4 doses) was administered.

Reporting of suspected adverse reactions

Reporting suspected adverse reactions after authorization of the medicinal product is important. It allows continued monitoring of the benefit/risk balance of the medicinal product. Healthcare professionals are asked to report any suspected adverse reactions (see details below).

14Ireland

HPRA
Pharmacovigi
lance
Website:
www.hpra.ie

15Malta

ADR Reporting

Website: www.medicinesauthority.gov.mt/adrportal

164.9 Overdose

There are limited data available on overdose with RoActemra. One case of accidental overdose was reported in which a patient with multiple myeloma received a single dose of 40 mg/kg. No adverse reactions were observed.

No serious adverse reactions were observed in healthy volunteers who received a single dose up to 28 mg/kg, although dose limiting neutropenia was observed.

17 Paediatric population

No case of an overdose in the paediatric population has been observed.

5. PHARMACOLOGICAL PROPERTIES

5.1 Pharmacodynamic properties

Pharmacotherapeutic group: Immunosuppressants, Interleukin inhibitors;

ATC code: L04AC07. Mechanism of action

Tocilizumab binds specifically to both soluble and membrane-bound IL-6 receptors (sIL-6R and

mIL-6R). Tocilizumab has been shown to inhibit sIL-6R and mIL-6R-mediated signalling. IL-6 is a pleiotropic pro-inflammatory cytokine produced by a variety of cell types including T- and B-cells, monocytes and fibroblasts. IL-6 is involved in diverse physiological processes such as T-cell activation, induction of immunoglobulin secretion, induction of hepatic acute phase protein synthesis and stimulation of haemopoiesis. IL-6 has been implicated in the pathogenesis of diseases including inflammatory diseases, osteoporosis and neoplasia.

RA Patients Pharmacodynamic effects

In clinical studies with tocilizumab, rapid decreases in CRP, erythrocyte sedimentation rate (ESR), serum amyloid A (SAA) and fibrinogen were observed. Consistent with the effect on acute phase reactants, treatment with tocilizumab was associated with reduction in platelet count within the normal range. Increases in haemoglobin levels were observed, through tocilizumab decreasing the IL-6 driven effects on hepcidin production to increase iron availability. In tocilizumab-treated patients, decreases in the levels of CRP to within normal ranges were seen as early as week 2, with decreases maintained while on treatment.

In healthy subjects administered tocilizumab in doses from 2 to 28 mg/kg, absolute neutrophil counts decreased to their lowest 3 to 5 days following administration. Thereafter, neutrophils recovered towards baseline in a dose dependent manner. Rheumatoid arthritis patients demonstrated a similar pattern of absolute neutrophil counts following tocilizumab administration (see section 4.8).

Clinical efficacy and safety

The efficacy of tocilizumab in alleviating the signs and symptoms of RA was assessed in five randomised, double-blind, multi-centre studies. Studies I-V enrolled patients ≥ 18 years of age with active RA diagnosed according to the American College of Rheumatology (ACR) criteria and who had at least eight tender and six swollen joints at baseline.

In Study I, tocilizumab was administered intravenously every four weeks as monotherapy. In Studies II, III and V, tocilizumab was administered intravenously every four weeks in combination with MTX vs. placebo and MTX. In Study IV, tocilizumab was administered intravenously every 4 weeks in combination with other DMARDs vs. placebo and other DMARDs. The primary endpoint for each of the five studies was the proportion of patients who achieved an ACR 20 response at week 24.

Study I evaluated 673 patients who had not been treated with MTX within six months prior to randomisation and who had not discontinued previous MTX treatment as a result of clinically important toxic effects or lack of response. The majority (67%) of patients were MTX-naïve. Doses of 8 mg/kg of tocilizumab were given every four weeks as monotherapy. The comparator group was weekly MTX (dose titrated from 7.5 mg to a maximum of 20 mg weekly over an eight week period).

Study II, a two year study with planned analyses at week 24, week 52 and week 104, evaluated 1,196 patients who had an inadequate clinical response to MTX. Doses of 4 or 8 mg/kg of tocilizumab or placebo were given every four weeks as blinded therapy for 52 weeks in combination with stable MTX (10 mg to 25 mg weekly). After week 52, all patients could receive open-label treatment with tocilizumab 8 mg/kg. Of the patients who completed the study who were originally randomised to placebo + MTX, 86% received open-label tocilizumab 8 mg/kg in year 2. The primary endpoint at week 24 was the proportion of patients who achieved an ACR 20 response. At week 52 and week 104 the co-primary endpoints were prevention of joint damage and improvement in physical function.

Study III evaluated 623 patients who had an inadequate clinical response to MTX. Doses of 4 or

8 mg/kg tocilizumab or placebo were given every four weeks, in combination with stable MTX (10 mg to 25 mg weekly).

Study IV evaluated 1,220 patients who had an inadequate response to their existing rheumatologic therapy, including one or more DMARDs. Doses of 8 mg/kg tocilizumab or placebo were given every four weeks in combination with stable DMARDs.

Study V evaluated 499 patients who had an inadequate clinical response or were intolerant to one or more TNF antagonist therapies. The TNF antagonist therapy was discontinued prior to randomisation. Doses of 4 or 8 mg/kg tocilizumab or

placebo were given every four weeks in combination with stable MTX (10 mg to 25 mg weekly).

Clinical response

In all studies, patients treated with tocilizumab 8 mg/kg had statistically significant higher ACR 20, 50, 70 response rates at 6 months compared to control (Table 3). In study I, superiority of tocilizumab 8 mg/kg was demonstrated against the active comparator MTX.

The treatment effect was similar in patients independent of rheumatoid factor status, age, gender, race, number of prior treatments or disease status. Time to onset was rapid (as early as week 2) and the magnitude of response continued to improve with duration of treatment. Continued durable responses were seen for over 3 years in the open label extension studies I-V.

In patients treated with tocilizumab 8 mg/kg, significant improvements were noted on all individual components of the ACR response including: tender and swollen joint counts; patients and physician global assessment; disability index scores; pain assessment and CRP compared to patients receiving placebo plus MTX or other DMARDs in all studies.

Patients in studies I – V had a mean Disease Activity Score (DAS28) of 6.5–6.8 at baseline. Significant reduction in DAS28 from baseline (mean improvement) of 3.1–3.4 were observed in tocilizumab-treated patients compared to control patients (1.3-2.1). The proportion of patients achieving a DAS28 clinical remission (DAS28 < 2.6) was significantly higher in patients receiving tocilizumab (28–34%) compared to 1–12% of control patients at 24 weeks. In study II, 65% of patients achieved a DAS28 < 2.6 at week 104 compared to 48% at 52 weeks and 33% of patients at week 24.

In a pooled analysis of studies II, III and IV, the proportion of patients achieving an ACR 20, 50 and 70 response was significantly higher (59% vs. 50%, 37% vs. 27%, 18% vs. 11%, respectively) in the tocilizumab 8 mg/kg plus DMARD vs. the tocilizumab 4 mg/kg plus DMARD group (p< 0.03).

Similarly the proportion of patients achieving a DAS28 remission (DAS28 < 2.6) was significantly higher (31% vs. 16% respectively) in patients receiving tocilizumab 8 mg/kg plus DMARD than in patients receiving tocilizumab 4 mg/kg plus DMARD (p< 0.0001).

Table 3. ACR responses in placebo-/MTX-/DMARDs-controlled studies (% patients)

	Study I AMBITION		Study II LITHE		Study III OPTION		Study IV TOWARD		Study V RADIATE	
Week	TCZ 8 mg/kg	MTX	TCZ 8 mg/kg + MTX	PBO + MTX	TCZ 8 mg/kg + MTX	PBO + MTX	TCZ 8 mg/kg + DMARD	PBO + DMARD	TCZ 8 mg/kg + MTX	PBO + MTX

	N = 28	N = 28	N = 39	N = 39	N = 20	N = 20	N = 80	N = 41	N = 17	N = 15
**ACR 20**										
24	70%***	52%	56%***	27%	59%***	26%	61%***	24%	50%***	10%
52			56%***	25%						
**ACR 50**										
24	44%**	33%	32%***	10%	44%***	11%	38%***	9%	29%***	4%
52			36%***	10%						
**ACR 70**										
24	28%**	15%	13%***	2%	22%***	2%	21%***	3%	12%**	1%
52			20%***	4%						

TCZ - Tocilizumab

MTX - Methotrexate

PBO - Placebo

DMARD - Disease modifying anti-rheumatic drug

*** - p< 0.01, TCZ vs. PBO + MTX/DMARD*

**** - p< 0.0001, TCZ vs. PBO + MTX/DMARD*

Major Clinical Response

After 2 years of treatment with tocilizumab plus MTX, 14% of patients achieved a major clinical response (maintenance of an ACR70 response for 24 weeks or more).

Radiographic response

In Study II, in patients with an inadequate response to MTX, inhibition of structural joint damage was assessed radiographically and expressed as change in modified Sharp score and its components, the erosion score and joint space narrowing score. Inhibition of joint structural damage was shown with significantly less radiographic progression in patients receiving tocilizumab compared to control (Table 4).

In the open-label extension of Study II the inhibition of progression of structural joint damage in tocilizumab plus MTX-treated patients was maintained in the second year of treatment. The mean change from baseline at week 104 in total Sharp-Genant score was significantly lower for patients randomised to tocilizumab 8 mg/kg plus MTX (p<0.0001) compared with patients who were randomised to placebo plus MTX.

Table 4. Radiographic mean changes over 52 weeks in Study II

	**PBO + MTX (+ TCZ from week 24) N = 393**	**TCZ 8 mg/kg + MTX N = 398**
Total Sharp-Genant score	1.13	0.29*
Erosion score	0.71	0.17*
JSN score	0.42	0.12**

PBO - Placebo

MTX *- Methotrexate*
TCZ *- Tocilizumab*
JSN *- Joint space narrowing*
*** *- p≤ 0.0001, TCZ vs. PBO + MTX*
**** *- p< 0.005, TCZ vs. PBO + MTX*

Following 1 year of treatment with tocilizumab plus MTX, 85% of patients (n=348) had no progression of structural joint damage, as defined by a change in the Total Sharp Score of zero or less, compared with 67% of placebo plus MTX-treated patients (n=290) (p ≤ 0.001). This remained consistent following 2 years of treatment (83%; n=353). Ninety three percent (93%; n=271) of patients had no progression between week 52 and week 104.

Health-related and quality of life outcomes

Tocilizumab-treated patients reported an improvement in all patient-reported outcomes (Health Assessment Questionnaire Disability Index - HAQ-DI), Short Form-36 and Functional Assessment of Chronic Illness Therapy questionnaires. Statistically significant improvements in HAQ-DI scores were observed in patients treated with RoActemra compared with patients treated with DMARDs. During the open-label period of Study II, the improvement in physical function has been maintained for up to 2 years. At Week 52, the mean change in HAQ-DI was -0.58 in the tocilizumab 8 mg/kg plus MTX group compared with -0.39 in the placebo + MTX group. The mean change in HAQ-DI was maintained at Week 104 in the tocilizumab 8 mg/kg plus MTX group (-0.61).

Haemoglobin levels

Statistically significant improvements in haemoglobin levels were observed with tocilizumab compared with DMARDs (p< 0.0001) at week 24. Mean haemoglobin levels increased by week 2 and remained within normal range through to week 24.

Tocilizumab versus adalimumab in monotherapy

Study VI (WA19924), a 24 week double-blinded study that compared tocilizumab monotherapy with adalimumab monotherapy, evaluated 326 patients with RA who were intolerant of MTX or where continued treatment with MTX was considered inappropriate (including MTX inadequate responders). Patients in the tocilizumab arm received an intravenous (IV) infusion of tocilizumab (8 mg/kg) every 4 weeks (q4w) and a subcutaneous (SC) placebo injection every 2 weeks (q2w). Patients in the adalimumab arm received an adalimumab SC injection (40 mg) q2w plus an IV placebo infusion q4w. A statistically significant superior treatment effect was seen in favour of tocilizumab over adalimumab in control of disease activity from baseline to week 24 for the primary endpoint of change in DAS28 and for all secondary endpoints (Table 5).

Table 5: Efficacy Results for Study VI (WA19924)

	ADA + Placebo (IV) N = 162	TCZ + Placebo (SC) N = 163	p-value[(a)]
**Primary Endpoint - Mean Change from baseline at Week 24**			
**DAS28 (adjusted mean)**	**-1.8**	**-3.3**	
**Difference in adjusted mean (95% CI)**	**-1.5 (-1.8, -1.1)**		**<0.0001**
**Secondary Endpoints - Percentage of Responders at Week 24 [(b)]**			
DAS28 < 2.6, n (%)	17	65 (39.9)	<0.0001

[a]p value is adjusted for region and duration of RA for all endpoints and additionally baseline value for all continuous endpoints.

[b] Non-responder Imputation used for missing data. Multiplicity controlled using Bonferroni-Holm Procedure

The overall clinical adverse event profile was similar between tocilizumab and adalimumab. The proportion of patients with serious adverse events was balanced between the treatment groups (tocilizumab 11.7% vs. adalimumab 9.9%). The types of adverse drug reactions in the tocilizumab arm were consistent with the known safety profile of tocilizumab and adverse drug reactions were reported at a similar frequency compared with Table 1. A higher incidence of infections and infestations was reported in the tocilizumab arm (48% vs. 42%), with no difference in the incidence of serious infections (3.1%). Both study treatments induced the same pattern of changes in laboratory safety parameters (decreases in neutrophil and platelet counts, increases in ALT, AST and lipids), however, the magnitude of change and the frequency of marked abnormalities was higher with tocilizumab compared with adalimumab. Four (2.5%) patients in the tocilizumab arm and two (1.2%) patients in the adalimumab arm experienced CTC grade 3 or 4 neutrophil count decreases. Eleven (6.8%) patients in the tocilizumab arm and five (3.1%) patients in the adalimumab arm experienced ALT increases of CTC grade 2 or higher. The mean LDL increase from baseline was 0.64 mmol/L

(25 mg/dL) for patients in the tocilizumab arm and 0.19 mmol/L (7 mg/dL) for patients in the adalimumab arm. The safety observed in the tocilizumab arm was consistent with the known safety profile of tocilizumab and no new or unexpected adverse drug reactions were observed (see Table 1).

MTX naïve, Early RA

Study VII (WA19926), a 2 year study with the planned primary analysis at week 52 evaluated 1162 MTX-naïve adult patients with moderate to severe, active early RA (mean disease duration ≤ 6 months). Approximately 20% of patients had received prior treatment with DMARDs other than MTX. This study evaluated the efficacy of IV tocilizumab 4 or 8 mg/kg every 4 weeks/MTX combination therapy, IV tocilizumab 8 mg/kg monotherapy and MTX monotherapy in reducing

the signs and symptoms and rate of progression of joint damage for 104 weeks. The primary endpoint was the proportion of patients achieving DAS28 remission (DAS28 < 2.6) at week 24. A significantly higher proportion of patients in the tocilizumab 8 mg/kg + MTX and tocilizumab monotherapy groups met the primary endpoint compared with MTX alone. The tocilizumab 8 mg/kg + MTX group also showed statistically significant results across the key secondary endpoints. Numerically greater responses compared with MTX alone were observed in the tocilizumab 8 mg/kg monotherapy group in all secondary endpoints, including radiographic endpoints. In this study, ACR/EULAR remission (Boolean and Index) were also analysed as pre-specified exploratory endpoints, with higher responses observed in the tocilizumab groups. The results from study VII are shown in Table 6.

Table 6: Efficacy Results for Study VII (WA19926) on MTX-naïve, early RA patients

			TCZ 8 mg/kg + + MTX N=290	TCZ 8 mg/kg + placebo N=292	TCZ 4 mg/kg + MTX N=288	Placebo MTX N=287
			**Primary Endpoint**			
DAS28 Remission						
	Week 24	n (%)	130 (44.8)***	113 (38.7)***	92 (31.9)	43 (15.0)
			**Key Secondary Endpoints**			
DAS 28 remission						
	Week 52	n (%),	142 (49.0)***	115 (39.4)	98 (34.0)	56 (19.5)
ACR						
	Week 24	ACR20, n (%)	216 (74.5)*	205 (70.2)	212 [illegible]	187 [illegible]
		ACR50, n (%)	165 (56.9)**	139 (47.6)	138 [illegible]	124 [illegible]
		ACR70, n (%)	112 (38.6)**	88 (30.1)	100 [illegible]	73 (25.4)
	Week 52	ACR20, n (%)	195 (67.2)*	184 (63.0)	181 [illegible]	164 [illegible]
		ACR50, n (%)	162 (55.9)**	144 (49.3)	151 [illegible]	117 [illegible]
		ACR70, n (%)	125 (43.1)**	105 (36.0)	107 [illegible]	83 (28.9)
HAQ-DI (adjusted mean change from baseline)						
	Week [illegible]		-0.81*	-0.67	-0.75	-0.64
			**Radiographic Endpoints (mean change from baseline)**			
	Week 52	mTSS	0.08***	0.26	0.42	1.14
		Erosion Score	0.05**	0.15	0.25	0.63
		JSN	0.03	0.11	0.17	0.51
		Radiographic Non-Progression n (%) (change from baseline in mTSS of ≤0)	226 (83)‡	226 (82)‡	211 (79)	194 (73)
			**Exploratory Endpoints**			
		Week 24: ACR/EULAR Boolean Remission, n	47 (18.4) ‡	38 (14.2)	43 (16.7) ‡	25 (10.0)
		(%) ACR/EULAR Index Remission,	73 (28.5) ‡	60 (22.6)	58 (22.6)	41 (16.4)
		n (%)	59 (25.7) ‡	43 (18.7)	48 (21.1)	34 (15.5)
		Week 52: ACR/EULAR Boolean Remission, n [illegible]	83 (36.1) ‡	69 (30.0)	66 (29.3)	49 (22.4)

mTSS - *modified Total Sharp Score*

JSN - *Joint space narrowing*

All efficacy comparisons vs Placebo + MTX. ***p≤0.0001; **p<0.001; *p<0.05;

‡p-value < 0.05 vs. Placebo + MTX, but endpoint was exploratory (not included in the hierarchy of statistical testing and has therefore not been controlled for multiplicity)

<u>Paediatric population</u>

sJIA Patients

Clinical efficacy

The efficacy of tocilizumab for the treatment of active sJIA was assessed in a 12 week randomised, double blind, placebo-controlled, parallel group, two arm study. Patients included in the trial had a total disease duration of at least 6 months and active disease but were not experiencing an acute flare requiring corticosteroid doses of more than 0.5 mg/kg prednisone equivalent. Efficacy for the treatment of macrophage activation syndrome has not been investigated.

Patients (treated with or without MTX) were randomised (tocilizumab:placebo = 2:1) to one of two treatment groups, 75 patients received tocilizumab infusions every two weeks, either 8 mg/kg for patients ≥ 30 kg or 12 mg/kg for patients < 30 kg and 37 patients were assigned to receiving placebo infusions every two weeks. Corticosteroid tapering was permitted from week six for patients who achieved a JIA ACR70 response. After 12 weeks or at the time of escape, due to disease worsening, patients were treated in the open label phase at weight appropriate dosing.

Clinical response

The primary endpoint was the proportion of patients with at least 30% improvement in the JIA ACR core set (JIA ACR30 response) at week 12 and absence of fever (no temperature recording ≥ 37.5°C in the preceding 7 days). Eighty five percent (64/75) of tocilizumab treated patients and 24.3% (9/37) of placebo treated patients achieved this endpoint. These proportions were highly significantly different (p<0.0001).

The percent of patients achieving JIA ACR 30, 50, 70 and 90 responses are shown in Table 7.

Table 7. JIA ACR response rates at week 12 (% patients)

Response Rate	Tocilizuma b N = 75	Placeb o N =
JIA ACR 30	90.7%	24.3%
JIA ACR 50	85.3%	10.8%
JIA ACR 70	70.7%	8.1%
JIA ACR 90	37.3%	5.4%

[1] *p<0.0001, tocilizumab vs. placebo*

Systemic Effects

In the tocilizumab treated patients, 85% who had fever due to sJIA at baseline were free of fever (no temperature recording ≥ 37.5°C in the preceding 14 days) at week 12 versus 21% of placebo patients (p<0.0001).

The adjusted mean change in the pain VAS after 12 weeks of tocilizumab treatment was a reduction of 41 points on a scale of 0 - 100 compared to a reduction of 1 for placebo patients (p<0.0001).

Corticosteroid Tapering

Patients achieving a JIA ACR70 response were permitted corticosteroid dose reduction. Seventeen (24%) tocilizumab treated patients versus 1 (3%) placebo patient were able to reduce their dose of corticosteroid by at least 20% without experiencing a subsequent JIA ACR30 flare or occurrence of systemic symptoms to week 12 (p=0.028). Reductions in corticosteroids continued, with 44 patients off oral corticosteroids at week 44, while maintaining JIA ACR responses.

Health related and quality of life outcomes

At week 12, the proportion of tocilizumab treated patients showing a minimally clinically important improvement in the Childhood Health Assessment Questionnaire – Disability Index (defined as an individual total score decrease of ≥ 0.13) was significantly higher than in placebo treated patients, 77% versus 19% (p<0.0001).

Laboratory Parameters

Fifty out of seventy five (67%) tocilizumab treated patients had a haemoglobin < LLN at baseline. Forty (80%) of these patients had an increase in their haemoglobin to within the normal range at week 12, in comparison to 2 out of 29 (7%) of placebo treated patients with haemoglobin < LLN at baseline (p<0.0001).

pJIA Patients

Clinical efficacy

The efficacy of tocilizumab was assessed in a three-part study WA19977 including an open-label extension in children with active pJIA. Part I consisted of a 16-week active tocilizumab treatment lead-in period (n=188) followed by Part II, a 24-week randomized double-blind placebo-controlled withdrawal period (n=163), followed by Part III, a 64-week open-label period. In Part 1, eligible patients ≥ 30 kg received tocilizumab at 8 mg/kg IV every 4 weeks for 4 doses. Patients < 30 kg were randomized 1:1 to receive either tocilizumab 8 mg/kg or 10 mg/kg IV every 4 weeks for 4 doses. Patients who completed Part I of the study and achieved at least a JIA ACR30 response at week 16 compared to baseline were eligible to enter the blinded withdrawal period (Part II) of the study. In Part II, patients were randomized to tocilizumab (same dose received in Part I) or placebo in a 1:1 ratio, stratified

by concurrent MTX use and concurrent corticosteroid use. Each patient continued in Part II of the study until Week 40 or until the patient satisfied JIA ACR30 flare criteria (relative to Week 16) and qualified for escape to tocilizumab therapy (same dose received in Part I).

Clinical response

The primary endpoint was the proportion of patients with a JIA ACR30 flare at week 40 relative to week 16. Forty eight percent (48.1%, 39/81) of the patients treated with placebo flared compared with 25.6% (21/82) of tocilizumab treated patients. These proportions were statistically significantly different (p=0.0024).

At the conclusion of Part I, JIA ACR 30/50/70/90 responses were 89.4%, 83.0%, 62.2%, and 26.1%, respectively.

During the withdrawal phase (Part II), the percentage of patients achieving JIA ACR 30, 50, and 70 responses at Week 40 relative to baseline are shown in Table 8. In this statistical analysis, patients who flared (and escaped to TCZ) during Part II or who withdrew, were classified as non-responders. An additional analyses of JIA ACR responses, considering observed data at Week 40, regardless of flare status, showed that by Week 40, 95.1% of patients who had received continuous TCZ therapy, had achieved JIA ACR30 or higher.

Table 8. JIA ACR Response Rates at Week 40 Relative to baseline (Percentage of Patients)

**Response Rate**	Tocilizuma<br>b N=82	Placeb<br>o
ACR 30	74.4%*	54.3%*
ACR 50	73.2%*	51.9%*
ACR 70	64.6%*	42.0%*

** p<0.01, tocilizumab vs. placebo*

The number of active joints was significantly reduced compared to baseline in patients receiving tocilizumab compared to placebo (adjusted mean changes of -14.3 vs -11.4, p=0.0435). The physician's global assessment of disease activity, as measured on a 0-100 mm scale, showed a greater reduction in disease activity for tocilizumab compared to placebo (adjusted mean changes of -45.2 mm vs -35.2 mm, p=0.0031).

The adjusted mean change in the pain VAS after 40 weeks of tocilizumab treatment was 32.4 mm on a 0-100 mm scale compared to a reduction of 22.3 mm for placebo patients (highly statistically significant; p=0.0076).

The ACR response rates were numerically lower for patients with prior biologic treatment as shown in Table 9 below.

Table 9. Number and Proportion of Patients with a JIA ACR30 Flare and Proportion of Patients with JIA ACR30/50/70/90 Responses at Week 40, by Previous Biologic Use (ITT Population - Study Part II)

	**Placebo**		**All TCZ**	
**Biologic Use**	**Yes (N = [illegible]**	**No (N = [illegible]**	**Yes (N = [illegible]**	**No (N = [illegible]**
JIA ACR30 Flare	18 (78.3)	21 (36.2)	12 (44.4)	9 (16.4)
JIA ACR30 Response	6 (26.1)	38 (65.5)	15 (55.6)	46 (83.6)
JIA ACR50 Response	5 (21.7)	37 (63.8)	14 (51.9)	46 (83.6)
JIA ACR70 Response	2 (8.7)	32 (55.2)	13 (48.1)	40 (72.7)
JIA ACR90 Response	2 (8.7)	17 (29.3)	5 (18.5)	32 (58.2)

Patients randomized to tocilizumab had fewer ACR30 flares and higher overall ACR responses than patients receiving placebo regardless of a history of prior biologic use.

CRS

The efficacy of RoActemra for the treatment of CRS was assessed in a retrospective analysis of data from clinical trials of CAR T-cell therapies (tisagenlecleucel and axicabtagene ciloleucel) for hematological malignancies. Evaluable patients had been treated with tocilizumab 8 mg/kg (12 mg/kg for patients < 30 kg) with or without additional high-dose corticosteroids for severe or life-threatening CRS; only the first episode of CRS was included in the analysis. The efficacy population for the tisagenlecleucel cohort included 28 males and 23 females (total 51 patients) of median age 17 years (range, 3–68 years). The median time from start of CRS to first dose of tocilizumab was 3 days (range, 0–18 days). Resolution of CRS was defined as lack of fever and off vasopressors for at least 24 hours. Patients were considered responders if CRS resolved within 14 days of the first dose of tocilizumab, if no more than 2 doses of RoActemra were needed, and no drugs other than RoActemra and corticosteroids were used for treatment. Thirty-nine patients (76.5%; 95% CI: 62.5%–87.2%) achieved a response. In an independent cohort of 15 patients (range: 9–75 years old) with axicabtagene ciloleucel-induced CRS, 53% responded.

The European Medicines Agency has waived the obligation to submit the results of studies with RoActemra in all subsets of the paediatric population in treatment of cytokine release syndrome associated with chimeric antigen receptor (CAR) T cell therapy.

5.2 Pharmacokinetic properties

RA Patients

Intravenous use

The pharmacokinetics of tocilizumab were determined using a population pharmacokinetic analysis on a database composed of 3552 RA patients treated with a one-hour infusion of 4 or 8 mg/kg tocilizumab every 4 weeks for 24 weeks or with 162 mg tocilizumab given subcutaneously either once a week or every other week for 24 weeks.

The following parameters (predicted mean ± SD) were estimated for a dose of 8 mg/kg tocilizumab given every 4 weeks: steady-state area under curve (AUC) = 38000 ± 13000 h µg/mL, trough concentration (C_{min}) = 15.9 ± 13.1 µg/mL and maximum concentration (C_{max}) = 182 ± 50.4 µg/mL, and the accumulation ratios for AUC and C_{max} were small, 1.32 and 1.09, respectively. The accumulation ratio was higher for C_{min} (2.49), which was expected based on the non-linear clearance contribution at lower concentrations. Steady-state was reached following the first administration for C_{max} and after 8 and 20 weeks for AUC and C_{min}, respectively. Tocilizumab AUC, C_{min} and C_{max} increased with increase of body weight. At body weight ≥ 100 kg, the predicted mean (± SD) steady- state AUC, C_{min} and C_{max} of tocilizumab were 50000 ± 16800 µg•h/mL, 24.4 ± 17.5 µg/mL, and 226 ±

50.3 µg/mL, respectively, which are higher than mean exposure values for the patient population (i.e. all body weights) reported above. The dose-response curve for tocilizumab flattens at higher exposure, resulting in smaller efficacy gains for each incremental increase in tocilizumab concentration such that clinically meaningful increases in efficacy were not demonstrated in patients treated with > 800 mg of tocilizumab. Therefore, tocilizumab doses exceeding 800 mg per infusion are not recommended (see section 4.2).

Distribution

In RA patients the central volume of distribution was 3.72, the peripheral volume of distribution was

3.35 resulting in a volume of distribution at steady state of 7.07.

Elimination

Following intravenous administration, tocilizumab undergoes biphasic elimination from the circulation. The total clearance of tocilizumab was concentration-dependent and is the sum of the linear and non-linear clearance. The linear clearance was estimated as a parameter in the population pharmacokinetic analysis and was 9.5 mL/h. The concentration-dependent non-linear clearance plays a major role at low tocilizumab concentrations. Once the non-linear clearance pathway is saturated, at higher tocilizumab concentrations, clearance is mainly determined by the linear clearance.

The $t_{1/2}$ of tocilizumab was concentration-dependent. At steady-state following a dose of 8 mg/kg every 4 weeks, the effective $t_{1/2}$ decreased with decreasing concentrations within a dosing interval from 18 days to 6 days.

Linearity

Pharmacokinetic parameters of tocilizumab did not change with time. A more than dose-proportional increase in the AUC and C_{min} was observed for doses of 4 and 8 mg/kg every 4 weeks. C_{max} increased dose-proportionally. At steady-

state, predicted AUC and C_{min} were 3.2 and 30 fold higher at 8 mg/kg as compared to 4 mg/kg, respectively.

Special populations

Renal impairment: **No formal study of the effect of renal impairment on the pharmacokinetics of tocilizumab has been conducted. Most of the patients in the population pharmacokinetic analysis had normal renal function or mild renal impairment. Mild renal impairment (creatinine clearance based on Cockcroft-Gault < 80 mL/min and ≥ 50 mL/min) did not impact the pharmacokinetics of tocilizumab.**

Hepatic impairment: **No formal study of the effect of hepatic impairment on the pharmacokinetics of tocilizumab has been conducted.**

Age, gender and ethnicity**: Population pharmacokinetic analyses in RA patients, showed that age, gender and ethnic origin did not affect the pharmacokinetics of tocilizumab.**

sJIA Patients:

The pharmacokinetics of tocilizumab were determined using a population pharmacokinetic analysis on a database composed of 140 sJIA patients treated with 8 mg/kg IV every 2 weeks (patients with a body weight ≥ 30 kg) 12 mg/kg IV every 2 weeks (patients with a body weight < 30 kg), 162 mg SC every week (patients weighing ≥ 30 kg), 162 mg SC every 10 days or every 2 weeks (patients weighing below 30 kg).

Table 10. Predicted mean ± SD PK parameters at steady-state after IV dosing in sJIA

**RoActemra PK Parameter**	**8 mg/kg Q2W ≥ 30 kg**	**12 mg/kg Q2W below 30 kg**
C_{max} (µg/mL)	256 ± 60.8	274 ± 63.8
C_{trough} (µg/mL)	69.7 ± 29.1	68.4 ± 30.0
C_{mean} (µg/mL)	119 ± 36.0	123 ± 36.0
Accumulation C_{max}	1.42	1.37
Accumulation C_{trough}	3.20	3.41
Accumulation C_{mean} or AUC_{τ}*	2.01	1.95

*τ = 2 weeks for IV regimens

After IV dosing, approximately 90% of the steady-state was reached by week 8 for both the 12 mg/kg (BW < 30 kg) and 8 mg/kg Q2W (BW ≥ 30 kg) regimens.

In sJIA patients, the central volume of distribution was 1.87 L and the peripheral volume of distribution was 2.14 L resulting in a volume of distribution at a steady state of 4.01 L. The linear clearance estimated as a parameter in the population pharmacokinetic analysis, was 5.7 mL/h.

The half life of tocilizumab in sJIA patients is up to 16 days for the two body weight categories (8 mg/kg for body weight ≥ 30 kg or 12 mg/kg for body weight < 30 kg) at week 12.

pJIA Patients:

The pharmacokinetics of tocilizumab in pJIA patients was characterized by a population pharmacokinetic analysis which included 237 patients who were treated with 8 mg/kg IV every 4 weeks (patients weighing ≥ 30 kg), 10 mg/kg IV every 4 weeks (patients weighing below 30 kg), 162 mg SC every 2 weeks (patients weighing ≥ 30 kg), or 162 mg SC every 3 weeks (patients weighing below 30 kg).

Table 11. Predicted mean ± SD PK parameters at steady-state after IV dosing in pJIA

**RoActemra PK Parameter**	**8 mg/kg Q4W ≥ 30 kg**	**10 mg/kg Q4W below 30 kg**
C_{max} (µg/mL)	183 ± 42.3	168 ± 24.8
C_{trough} (µg/mL)	6.55 ± 7.93	1.47 ± 2.44
C_{mean} (µg/mL)	42.2 ± 13.4	31.6 ± 7.84
Accumulation C_{max}	1.04	1.01
Accumulation C_{trough}	2.22	1.43
Accumulation C_{mean} or $AUC_{τ}$*	1.16	1.05

*τ = 4 weeks for IV regimens

After IV dosing, approximately 90% of the steady-state was reached by week 12 for the 10 mg/kg (BW < 30 kg), and by week 16 for the 8 mg/kg (BW ≥ 30 kg) dose.

The half life of tocilizumab in pJIA patients is up to 16 days for the two body weight categories

(8 mg/kg for body weight ≥ 30 kg or 10 mg/kg for body weight < 30 kg) during a dosing interval at steady state.

185.3 Preclinical safety data

Non-clinical data reveal no special hazard for humans based on conventional studies of safety pharmacology, repeated dose toxicity and genotoxicity.

Carcinogenicity studies were not performed because IgG1 monoclonal antibodies are not deemed to have intrinsic carcinogenic potential.

Available non-clinical data demonstrated the effect of IL-6 on malignant progression and apoptosis resistance to various cancer types. This data does not suggest a relevant risk for cancer initiation and progression under

tocilizumab treatment. Additionally, proliferative lesions were not observed in a 6-month chronic toxicity study in cynomolgus monkeys or in IL-6 deficient mice.

Available non-clinical data do not suggest an effect on fertility under tocilizumab treatment. Effects on endocrine active and reproductive system organs were not observed in a chronic cynomolgus monkey toxicity study and reproductive performance was not affected in IL-6 deficient mice. Tocilizumab administered to cynomolgus monkeys during early gestation, was observed to have no direct or indirect harmful effect on pregnancy or embryonal-foetal development. However, a slight increase in abortion/embryonal-foetal death was observed with high systemic exposure (> 100 x human exposure) in the 50 mg/kg/day high-dose group compared to placebo and other low-dose groups. Although IL-6 does not seem to be a critical cytokine for foetal growth or the immunological control of the maternal/foetal interface, a relation of this finding to tocilizumab cannot be excluded.

Treatment with a murine analogue did not exert toxicity in juvenile mice. In particular, there was no impairment of skeletal growth, immune function and sexual maturation.

6. PHARMACEUTICAL PARTICULARS

6.1 List of excipients

Su cro se Pol ys orb ate 80

Disodium phosphate dodecahydrate Sodium dihydrogen phosphate dihydrate Water for injections

6.2 Incompatibilities

This medicinal product must not be mixed with other medicinal products except those mentioned in section 6.6.

6.3 Shelf life

Unopened vial: 30 months

***Diluted product*: After dilution, the prepared solution for infusion is physically and chemically stable in sodium chloride 9 mg/mL (0.9%) solution for injection at 30ºC for 24 hours.**

From a microbiological point of view, the prepared solution for infusion should be used immediately. If not used immediately, in use storage times and conditions prior to use are the responsibility of the user and would normally not be longer than 24 hours at 2°C–8°C, unless dilution has taken place in controlled and validated aseptic conditions.

6.4 Special precautions for storage

Store vials in a refrigerator (2°C–8°C). Do not freeze.

Keep the vial(s) in the outer carton in order to protect from light.

For storage conditions of the diluted medicinal product see section 6.3.

6.5 Nature and contents of container

RoActemra is supplied in a vial (type I glass) with a stopper (butyl rubber) containing 4 mL, 10 mL or 20 mL concentrate. Pack sizes of 1 and 4 vials.

Not all pack sizes may be marketed.

6.6 Special precautions for disposal and other handling

Instructions for dilution prior to administration

Parenteral medicinal products should be inspected visually for particulate matter or discolouration prior to administration. Only solutions which are clear to opalescent, colourless to pale yellow and free of visible particles should be diluted.

RA and CRS Patients (≥ 30 kg)

Withdraw a volume of sterile, non-pyrogenic sodium chloride 9 mg/mL (0.9%) solution for injection from a 100 mL infusion bag, equal to the volume of RoActemra concentrate required for the patients dose, under aseptic conditions. The required amount of RoActemra concentrate (0.4 mL/kg) should be withdrawn from the vial and placed in the 100 mL infusion bag. This should be a final volume of

100 mL. To mix the solution, gently invert the infusion bag

to avoid foaming. Use in the paediatric population

sJIA, pJIA and CRS Patients ≥ 30 kg

Withdraw a volume of sterile, non-pyrogenic sodium chloride 9 mg/mL (0.9%) solution for injection from a 100 mL infusion bag, equal to the volume of RoActemra concentrate required for the patients dose, under aseptic conditions. The required amount of RoActemra concentrate (0.4 mL/kg) should be withdrawn from the vial and placed in the 100 mL infusion bag. This should be a final volume of

100 mL. To mix the solution, gently invert the infusion bag to avoid foaming.

sJIA and CRS Patients < 30 kg

Withdraw a volume of sterile, non-pyrogenic sodium chloride 9 mg/mL (0.9%) solution for injection from a 50 mL infusion bag, equal to the volume of RoActemra concentrate required for the patients dose, under aseptic conditions. The required amount of RoActemra concentrate (0.6 mL/kg) should be withdrawn from the vial and placed in the 50 mL infusion bag. This should be a final volume of

50 mL. To mix the solution, gently invert the infusion bag to avoid foaming.

pJIA Patients < 30 kg

Withdraw a volume of sterile, non-pyrogenic sodium chloride 9 mg/mL (0.9%) solution for injection from a 50 mL infusion bag, equal to the volume of RoActemra concentrate required for the patients dose, under aseptic conditions. The required amount of RoActemra concentrate (0.5 mL/kg) should be withdrawn from the vial and placed in the 50 mL infusion bag. This should be a final volume of

50 mL. To mix the solution, gently invert the infusion bag to avoid foaming.

RoActemra is for single-use only.

Any unused product or waste material should be disposed of in accordance with local requirements.

7. MARKETING AUTHORISATION HOLDER

Roche
Registration
GmbH Emil-
Barell-
Strasse 1

79639 Grenzach-Wyhlen Germany

8. MARKETING AUTHORISATION NUMBER(S)

EU/1/08/492/001
EU/1/08/492/002

EU/1/08/492/003
EU/1/08/492/004
EU/1/08/492/005
EU/1/08/492/006

9. DATE OF FIRST AUTHORISATION/DATE OF LATEST RENEWAL

Date of first authorisation: 16 January 2009 Date of last renewal: 25 September 2013

10. DATE OF REVISION OF THE TEXT

17 October 2019

Detailed information on this medicinal product is available on the website of the European Medicines Agency http://www.ema.europa.eu/.

Appendix B- Product Labelling

One vial of RoAtemra 20mg/mL Concentrate for solution for infusion.

Study Reference Number: UCDCRC/20/02

Patient Number:______________________________

Investigator:_________________________________

Site Address:________________________________

Contact Number:____________________________

Expiry:_____________________________________

Batch Number:______________________________

Directions for use: for administration as directed by study investigator for protocol UCDCRC/20/02.

Storage Conditions: Store in a refrigerator (2°C - 8°C). Do not freeze. Keep protected from light.

For Clinical Trial Use Only

Appendix C- Sample Storage

As part of this study we will collect and store subject's biological samples:

- Plasma collection: EDTA venipuncture tube: 4 x 9 ml
- Serum collection: 2 x 7.5 ml STT or equivalent
- Urine: Specimen collection container (10-60 ml). A minimum of 8 ml of urine
- Stool: fresh stool collected in a clean collection container (10-60mls).
- Respiratory sample: a sputum sample or nasopharyngeal aspirate placed in viral transport media

These samples will be stored in a secure biobank facility at the UCD Centre for Experimental Host Pathogen Research (CEPHR) for current and future medical research.

A Laboratory requisition form must be completed and sent along with the samples to the Central lab.

Sample(s) will be securely stored for up to 15 years. At this time, the sample(s) will be physically destroyed.

1
- Call courier to arrange pickup

2
- Ensure that all samples are clearly labelled and any central lab paperwork has been completed

3
- Package samples in bubble wrap pouches provided or wrap in bubble wrap casing

4
- Place absorbent material provided in the bottom of the biohazard bottle and pack samples into the bottle - a 50ml limit applies per bottle.
- Ensure bottle is clearly labelled with infectious disease sticker

5
- Place the bio-bottle in the outer cardboard package and label clearly with infectious and dry-ice stickers

6
- Once all bottles are packaged, ensure the shipment of dangerous good form is completed.
- The courier company will have prepared most of this form.
- Complete the volume and signature/place/date section

7
- Complete courier delivery paperwork

8
- The sample is now ready for collection

Flow chart for Packaging and Shipment of samples

Appendix D- Laboratory Reference Ranges

Test	Abbott Ref Range	MMUH Ref Range	Comments: Source of References Ranges (2.5-97.5th) is mainly MMUH unless otherwise stated
**Chemistry**			
ALT	0-55 u/L	**11 – 58**	
AST	5-34 U/L	**19-42**	
Albumin P	35-50 g/L (34-48 IF >60)	**35-50**	Kit insert
ALK	40-150 U/L (>20Y M, >15Y F)	**30-130**	Pathology Harmonization
Amylase	23-96 U/L	**28-97**	
Bil Total	3.4-20.5 umol/L	**5-24**	
Bilirubin direct	0-8.6 umol/L (25-66 Y)	**2.4-8.1**	
Chol	NA	**<5.18**	<5.18 is optimal (NCEP guidelines)
Chol HDL	NA	**>1.55**	>1.55 is optimal <1.04 confers CV risk (NCEP guidelines)
Chol LDL	NA (mmol/L)	**<2.6**	<2.60 is optimal (NCEP guidelines)
CK	30-200 (M) + 29-168 (F) U/L		
	**FEMALE**	**33-208**	
	**MALE**	**44-272**	
Chloride	101-110 mmol/L	**95-108**	Pathology Harmonization
C02	22-29 mmol/L	**22-29**	Pathology Harmonization (= Abbott)
Creatinine (enz)	64-104 (M), 49-90 (F) umol/L		
	**FEMALE**	**46-86**	
	**MALE**	**65-107**	
CRP	<5 mg/L	**<7**	
GGT	12-64 (M) and 9-36 (F) U/L		
	**FEMALE**	**8-53**	
	**MALE**	**11-67**	
Glucose	3.89-5.83 mmol/L	**≤6.0**	Use WHO guideline: Normal ≤6.0 mmol/L(Fasting)
Iron (non-fasting)	5.5-25.78 (M), 4.48-27.92 (F) umol/L		
	**FEMALE**	**6-33**	
	**MALE**	**12-32**	
K	3.5-5 mmol/L	**3.5-5.3**	Pathology Harmonization
	(wider K phoning range for Dial/HDU/ITU):		

Lactate	**0.5-2.2 nmol/L**	**0.5-2.2**	Kit insert
LDH	125-243 U/L	**120-220**	
Mg	0.66-1.07 mmol/L	**0.70-1.00**	Pathology Harmonization
Na	137-144 mmol/L	**133-146**	Pathology Harmonization
Osmolality (Serum)		**285-295**	
Phos	0.74-1.52 mmol/L	**0.74-1.52**	Kit insert
Protein (Total)	64-83 g/L (ambulatory)	**63-80**	Pathology Harmonization
Transferrin	**1.8-3.82** (14-60Y) **+ 1.73-3.60** (60-80Y**) (FEMALE**	**1.93-3.08**	g/L
	**1.74-3.64** (14-60Y) + **1.63-3.44** (60-80Y) **MALE**	**1.88-3.02**	g/L
Trig	<1.7 mmol/L = desirible	**<1.7**	<1.7 is optimal (NCEP guidelines)
Urea	2.5-9 mmol/L (incl. both sexes + </> 50y)	**2.8-8.6**	
Uric Acid	210-420 (M) + 150-350 (f) mmol/L<br>(180-385 - av of sexes)	**177-465**	
**Immunoassay:**			
AFP	1.09-8.04 ng/ml	**0.8-9.1**	
Anti-thyroglobulin	<4.11 IU/ml	**0.45-4.7**	10% CV = 4.11. Use for research but report Immulite and RIA on all samples
AntI-TPO	<5.61 IU/ml	**5.61**	10% CV = 5.61. Use 5.61
B12	189-883 pg/ml (ng/L)	**211-760**	
BETA-HCG	**<5 mIU/ml**	**<5**	Kit insert
BNP	<135 pg/ml	**<135**	Kit insert
CA-199	**3-37 u/ml (94.4%)**	**2-23**	
CA-15-3	**31.3 U/ml (99%F/98.5M)**	**0-31.3**	Kit insert
CA-125	**35 U/ml (94%F)**	**0-35**	Kit insert
CEA	**<5 ng/ml (93.5%)**	**0-5**	Kit insert
CKMB (Mass)	**3.4 AND 7.2 ng/ml (99% F AND M)**	**Total (range) + CKMB % but no range**	
Cortisol	101.2-535.7 nmol/L	**150-455 (AM sample)**	No range quoted for random or midnight
Ferritin	**21.81-274.66 (M)** + 4.63-204 ng/ml (f)		*=(ug/L)
	**FEMALE**	**8-247**	
	**MALE**	**22-275**	
Folate	7-31.4 ng/ml	**3.8-18.2**	=ug/L
Folate Red Cell	**235.1-788.5 ug/L**	**235-788**	Kit insert

FSH	0.95-11.95 mIU/ml (Males)<br>Female:<br>Follicular phase: 3.0-8.1 IU/L<br>Mid-cycle peak: 2.6-16.7 IU/l<br>Luteal phase: 1.4-5.5 IU/l	**1.4-10.8 (M)**	Kit insert quoted for females
freeT4	0.7-1.48 x 12.87 = 9-19 pmol/L	**9-20**	
freeT3	1.71-3.71 pg/ml x 1.536 = 2.6-5.7 pmol/L	**2.6-6.2**	
Insulin	NA (+ not specified)	**NA**	
i-PTH	15-68.3 pg/ml x 0.106 = 1.59-7.24 pmol/L	**1.89-7.61**	
LH	1.14-8.75 mIU/ml (male)<br>Female:<br>Follicular phase: 1.8-11.8 IU/l<br>Mid-cycle peak: 7.6-89.1 IU/l<br>Luteal phase: 0.6-14.0 IU/l	**1.39-6.48 (M)**	Kit insert quoted for females
Oestradiol	**40-161 pmol/L (Males)**	**40-161**	Kit insert
pmol/L	Follicular	**92-921**	Kit insert. Pre-menopausal
	Mid-Cycle	**139-2382**	Kit insert. Pre-menopausal
	Luteal	**92-1145**	Kit insert. Pre-menopausal
Progesterone	NA	**NA**	Revisit RCOG guidelines for source of assay providing diagnostic progesterone concentrations
Prolactin	3.56-19.4(m) 5.18-26.53 (f) ng/ml X21		
	**Female 109-557 mIU/L**	**116-585**	
	**Male 75-407 mIU/L**	**103-460**	
SHBG	13.5-71.4 (M) 19.8-155.2 nmol/L (F)	**35-118 (F)**	Established using an earlier cohort of MPH (Healthcheck) samples (Apr/May 2011)
		**13-56 (M)**	
TnI	NA: 0.013/0.033 ug/L f/m = 99% centile	**NA**	Use <0.03. 0.03-0.3. Correlate clinically. >0.3 suggests MI in clinically relevant circumstances
Total PSA	**M <49 + f = <4 ng/ml then % >4 increase from 2.5 to 7.3 for each decade**	**<4**	
TSH	0.35-4.94 uIU/ml	**0.35-4.94**	Kit insert

Appendix E - Instructions for measuring height and weight.

General guidelines:

Height and weight should be measured at relevant study visits under the following conditions:

Weight:

Subjects should be fasting for at least ten hours (other than small amounts of water) for all measurements of weight and body composition.

Subjects should be weighed, wearing only a hospital gown, underwear and socks. All other clothing, including shoes, should be removed.

Subjects should be asked to void before weight and body composition are measured.

Whenever possible, weight and body composition should not be measured during bouts of severe diarrhoea or other obvious disturbances of hydration status.

Subjects should not engage in strenuous exercise for 8 hours preceding the measurements because of its potential effect on hydration status.

Height:

This measure should be carefully performed using a standiometer, a measuring rod often found attached to scales, or other device that is carefully mounted and maintained throughout the study. If no designated equipment is available for measuring height, a tape measure or series of yardsticks should be carefully attached to a wall, with a zero end just touching the floor.

Shoes should be removed before height is measured.

For wall-mounted measuring devices, the subject should stand facing away from the device. They should be aligned so that the device runs up the middle of the body and standing with heels together and heels, buttocks and shoulders touching the wall. The subject should stand as straight as possible with chin resting on chest. Any horizontal measuring bar should be raised above the subject’s head and lowered until it just touches the head (the skull, not just the hair). It is important to make certain that the bar is completely horizontal. If the bar is at an angle greater or less than 90 degrees to the wall, the measurement of the height will be inaccurate.

Appendix F - Instructions for measuring hip and waist circumferences.

General guidelines:

Subjects should be dressed in underwear, socks and a hospital gown; all other clothing should be removed.

Use non-stretchable cloth or vinyl measuring tape that measures in centimetres or millimetres and is at least 1 cm in width.

Make sure the tape does not compress the tissues during the measurement.

Measuring tape should always be read at eye level.

All measurements should be performed three times and results recorded in centimetres to the nearest millimetre.

Measurement of Hip Circumference:

The subject should be standing erect but relaxed.

The subject should be encouraged not to hold in their stomach during the measurement.

Viewing the subject from the side, visually identify the widest width of the hip. The hospital gown may be held to conform to the subjects contour; the widest point is generally where there is maximal protuberance of the buttocks.

Measure circumference at that point, making sure the measuring tape is exactly parallel to the floor. Record the result in centimetres to the nearest millimetre.

Perform the measurements three times.

Measurement of Waist Circumference:

The subject should be standing erect but relaxed.

The subject should be encouraged not to hold in their stomach during the measurement.

All measurements should be made after the subject has exhaled.

Midwaist: Locate the upper border of the right iliac crest and measure the waist circumference at this level. The tape measure should be parallel to the floor.

Perform the measurements three times. Record the results in centimetres to the nearest millimetre.

Appendix G – Adverse Events and Serious Adverse Events.

Definition of an adverse event (AE).

An AE is any untoward medical occurrence in a patient or investigation subject administered a pharmaceutical product and which does not necessarily have a causal relationship with this treatment or investigations occurring as part of the study protocol.

An AE includes:

Exacerbation or increase in frequency or severity of a pre-existing illness.

Diagnosis of a condition occurring after administration of study drug, even if the subject may have experienced an episode in the past, occurring prior to the start of the study.

Persistent symptoms, present at baseline, that intensify after the start of the study.

An AE does not include:

Medical or surgical procedure (although a condition leading to surgical intervention may be an AE).

Pre-existing conditions that do not worsen after the start of the study.

Hospital admissions not related to an untoward medical occurrence (eg cosmetic surgery).

Definition of a Serious Adverse Event (SAE).

A SAE is any adverse event occurring at any dose that results in any of the following outcomes:

Death.

A life threatening adverse event.

Inpatient hospitalisation or prolongation of existing hospitalisation.

A disability/incapacity.

A congenital anomaly in the offspring of a subject who received drug.

Important medical events that may not result in death, be life threatening, or require hospitalisation may be considered a SAE when, based upon appropriate medical judgement, they may jeopardise the patient or subject and may require medical or surgical intervention to prevent one of the outcomes listed in this definition.

Abnormal Laboratory Assessments:

Abnormal laboratory assessments (including all scanning procedures) determined by the investigator as clinically significant, must be recorded as AEs or SAEs as defined in the above definitions. The investigator should exercise his/her medical and scientific judgement in deciding whether an abnormal laboratory finding or other abnormal assessment is clinically significant.

Appendix H - Declaration of Helsinki

52nd WMA General Assembly, Edinburgh, Scotland, October 2000

Ethical Principles for Medical Research Involving Human Subjects

HUMAN EXPERIMENTATION

In 1964, the World Medical Association drew up a code of ethics on human experimentation. This code, known as the Declaration of Helsinki, adopted by the 18th WMA General Assembly, Helsinki, Finland, June 1964 and amended by the 29th WMA General Assembly, Tokyo, Japan, October 1975, the 35th WMA General Assembly, Venice, Italy, October 1983, the 41st WMA General Assembly, Hong Kong, September 1989, the 48th WMA General Assembly, Somerset West, Republic of South Africa, October 1996 and the 52nd WMA General Assembly, Edinburgh, Scotland, October 2000 reads:

A. Introduction

(1) The World Medical Association has developed the Declaration of Helsinki as a statement of ethical principles to provide guidance to physicians and other participants in medical research involving human subjects. Medical research involving human subjects includes research on identifiable human material or identifiable data.

(2) It is the duty of the physician to promote and safeguard the health of the people. The physician's knowledge and conscience are dedicated to the fulfillment of this duty.

(3) The Declaration of Geneva of the World Medical Association binds the physician with the words, "The health of my patient will be my first consideration," and the International Code of Medical Ethics declares that, "A physician shall act only in the patient's interest when providing medical care which might have the effect of weakening the physical and mental condition of the patient."

(4) Medical progress is based on research which ultimately must rest in part on experimentation involving human subjects.

(5) In medical research on human subjects, considerations related to the well-being of the human subject should take precedence over the interests of science and society.

(6) The primary purpose of medical research involving human subjects is to improve prophylactic, diagnostic and therapeutic procedures and the understanding of the aetiology and pathogenesis of disease. Even the best proven prophylactic, diagnostic, and therapeutic methods must continuously be challenged through research for their effectiveness, efficiency, accessibility and quality.

(7) In current medical practice and in medical research, most prophylactic, diagnostic and therapeutic procedures involve risks and burdens.

(8) Medical research is subject to ethical standards that promote respect for all human beings and protect their health and rights. Some research populations are vulnerable and need special protection. The particular needs of the economically and medically disadvantaged must be recognized. Special attention is also required for those who cannot give or refuse consent for themselves, for those who may be subject to giving consent under duress, for those who will not benefit personally from the research and for those for whom the research is combined with care.

(9) Research Investigators should be aware of the ethical, legal and regulatory requirements for research on human subjects in their own countries as well as applicable international requirements. No national ethical, legal or regulatory requirement should be allowed to reduce or eliminate any of the protections for human subjects set forth in this Declaration.

B. Basic principles for all medical research

(10) It is the duty of the physician in medical research to protect the life, health, privacy, and dignity of the human subject.

(11) Medical research involving human subjects must conform to generally accepted scientific principles, be based on a thorough knowledge of the scientific literature, other relevant sources of information, and on adequate laboratory and, where appropriate, animal experimentation.

(12) Appropriate caution must be exercised in the conduct of research which may affect the environment, and the welfare of animals used for research must be respected.

(13) The design and performance of each experimental procedure involving human subjects should be clearly formulated in an experimental protocol. This protocol should be submitted for consideration, comment, guidance, and where appropriate, approval to a specially appointed ethical review committee, which must be independent of the investigator, the sponsor or any other kind of undue influence. This independent committee should be in conformity with the laws and regulations of the country in which the research experiment is performed. The committee has the right to monitor ongoing trials. The researcher has the obligation to provide monitoring information to the committee, especially any serious adverse events. The researcher should also submit to the committee, for review, information regarding funding, sponsors, institutional affiliations, other potential conflicts of interest and incentives for subjects.

(14) The research protocol should always contain a statement of the ethical considerations involved and should indicate that there is compliance with the principles enunciated in this Declaration.

(15) Medical research involving human subjects should be conducted only by scientifically qualified persons and under the supervision of a clinically competent medical person. The responsibility for the human subject must always rest with a medically qualified person and never rest on the subject of the research, even though the subject has given consent.

(16) Every medical research project involving human subjects should be preceded by careful assessment of predictable risks and burdens in comparison with foreseeable benefits to the subject or to others. This does not preclude the participation of healthy volunteers in medical research. The design of all studies should be publicly available.

(17) Physicians should abstain from engaging in research projects involving human subjects unless they are confident that the risks involved have been adequately assessed and can be satisfactorily managed. Physicians should cease any investigation if the risks are found to outweigh the potential benefits or if there is conclusive proof of positive and beneficial results.

(18) Medical research involving human subjects should only be conducted if the importance of the objective outweighs the inherent risks and burdens to the subject. This is especially important when the human subjects are healthy volunteers.

(19) Medical research is only justified if there is a reasonable likelihood that the populations in which the research is carried out stand to benefit from the results of the research.

(20) The subjects must be volunteers and informed participants in the research project.

(21) The right of research subjects to safeguard their integrity must always be respected. Every precaution should be taken to respect the privacy of the subject, the confidentiality of the patient's information and to minimize the impact of the study on the subject's physical and mental integrity and on the personality of the subject.

(22) In any research on human beings, each potential subject must be adequately informed of the aims, methods, sources of funding, any possible conflicts of interest, institutional affiliations of the researcher, the anticipated benefits and potential risks of the study and the discomfort it may entail. The subject should be informed of the right to abstain from participation in the study or to withdraw consent to participate at any time without reprisal. After ensuring that the subject has understood the information, the physician should then obtain the subject's freely-given informed consent, preferably

in writing. If the consent cannot be obtained in writing, the non-written consent must be formally documented and witnessed.

(23) When obtaining informed consent for the research project the physician should be particularly cautious if the subject is in a dependent relationship with the physician or may consent under duress. In that case the informed consent should be obtained by a well-informed physician who is not engaged in the investigation and who is completely independent of this relationship.

(24) For a research subject who is legally incompetent, physically or mentally incapable of giving consent or is a legally incompetent minor, the investigator must obtain informed consent from the legally authorized representative in accordance with applicable law. These groups should not be included in research unless the research is necessary to promote the health of the population represented and this research cannot instead be performed on legally competent persons.

(25) When a subject deemed legally incompetent, such as a minor child, is able to give assent to decisions about participation in research, the investigator must obtain that assent in addition to the consent of the legally authorized representative.

(26) Research on individuals from whom it is not possible to obtain consent, including proxy or advance consent, should be done only if the physical/mental condition that prevents obtaining informed consent is a necessary characteristic of the research population. The specific reasons for involving research subjects with a condition that renders them unable to give informed consent should be stated in the experimental protocol for consideration and approval of the review committee. The protocol should state that consent to remain in the research should be obtained as soon as possible from the individual or a legally authorized surrogate.

(27) Both authors and publishers have ethical obligations. In publication of the results of research, the investigators are obliged to preserve the accuracy of the results. Negative as well as positive results should be published or otherwise publicly available. Sources of funding, institutional affiliations and any possible conflicts of interest should be declared in the publication. Reports of experimentation not in accordance with the principles laid down in this Declaration should not be accepted for publication.

C. Medical research combined with professional care (clinical research)

(28) The physician may combine medical research with medical care, only to the extent that the research is justified by its potential prophylactic, diagnostic or therapeutic value. When medical research is combined with medical care, additional standards apply to protect the patients who are research subjects.

(29) The benefits, risks, burdens and effectiveness of a new method should be tested against those of the best current prophylactic, diagnostic, and therapeutic methods. This does not exclude the use of placebo, or no treatment, in studies where no proven prophylactic, diagnostic or therapeutic method exists.

(30) At the conclusion of the study, every patient entered into the study should be assured of access to the best proven prophylactic, diagnostic and therapeutic methods identified by the study.

(31) The physician should fully inform the patient which aspects of the care are related to the research. The refusal of a patient to participate in a study must never interfere with the patient-physician relationship.

(32) In the treatment of a patient, where proven prophylactic, diagnostic and therapeutic methods do not exist or have been ineffective, the physician, with informed consent from the patient, must be free to use unproven or new prophylactic, diagnostic and therapeutic measures, if in the physician's judgement it offers hope of saving life, re-establishing health or alleviating suffering. Where possible, these measures should be made the object of research, designed to evaluate their safety and efficacy. In all cases, new information should be recorded and, where appropriate, published. The other relevant guidelines of this Declaration should be followed.
